# Retrofitting passive cooling strategies to combat heat stress in the face of climate change: A case study of a ready-made garment factory in Dhaka, Bangladesh

**DOI:** 10.1016/j.enbuild.2023.112954

**Published:** 2023-05

**Authors:** Aaron J.E. Bach, Jean P. Palutikof, Fahim N. Tonmoy, James W. Smallcombe, Shannon Rutherford, Ashikur R. Joarder, Monir Hossain, Ollie Jay

**Affiliations:** a National Climate Change Adaptation Research Facility (NCCARF), Griffith University, Gold Coast, QLD, Australia; b Cities Research Institute, Griffith University, Gold Coast, QLD, Australia; c BMT Group, Brisbane, QLD, Australia; d Faculty of Medicine and Health, University of Sydney, Sydney, NSW, Australia; e Heat and Health Research Incubator, University of Sydney, Sydney, NSW, Australia; f School of Medicine and Dentistry, Griffith University, Gold Coast, QLD, Australia; g Department of Architecture, Bangladesh University of Engineering and Technology (BUET), Dhaka, Bangladesh

**Keywords:** Building modelling, Building simulation, Occupational health and safety, Wet-bulb globe temperature, Climate adaptation, Sustainable cooling

## Abstract

The ready-made garment industry is critical to the Bangladesh economy. There is an urgent need to improve current working conditions and build capacity for heat mitigation as conditions worsen due to climate change. We modelled a typical, mid-sized, non-air-conditioned factory in Bangladesh and simulated how the indoor thermal environment is altered by four rooftop retrofits (1. extensive green roof, 2. rooftop shading, 3. white cool roof, 4. insulated white cool roof) on present-day and future decades under different climate scenarios. Simulations showed that all strategies reduce indoor air temperatures by around 2 °C on average and reduce the number of present-day annual work-hours during which wetbulb globe temperature exceeds the standardised limits for moderate work rates by up to 603 h - the equivalent of 75 (8 h) working days per year. By 2050 under a high-emissions scenario, indoor conditions with a rooftop intervention are comparable to present-day conditions. To reduce the growing need for carbon-intensive air-conditioning, sustainable heat mitigation strategies need to be incorporated into a wider range of solutions at the individual, building, and urban level. The results presented here have implications for factory planning and retrofit design, and may inform policies targeting worker health, well-being, and productivity.

## 1 Introduction

Heat stress, and its impact on worker health and productivity, is a global problem spanning a range of industries, regions, and economies. Heat exposure reduces the physiological capacity of the human body to perform work relative to more temperate conditions and increases the risk of heat-related health disorders. The downstream impacts of occupational heat exposure are lower worker productivity [1–3], higher workplace injuries [4], higher worker attrition [5], loss of income [6], and a greater reliance upon a younger workforce [7,8]. To combat these problems, occupational health and safety standards have been established, predominantly in developed economies where collective industrial, institutional, and legislative importance has been placed on workplace safety.

Workplaces can implement a variety of evidence-based heat stress reduction and monitoring strategies at the individual level (e.g., personal cooling systems, rest strategies, improved hydration and altered clothing) and at the building level (e.g., alterations to building envelope materials, shading provisions, ventilation, air-conditioning) [9,10]. An essential component of occupational heat risk assessment is the measurement of key characteristics that define the thermal environment in order to generate composite heat stress indices such as wet-bulb globe temperature (WBGT). WBGT is a commonly used index in occupational health and safety with the potential to assess the severity of heat stress in workers [11]. It combines three temperature measurements (dry-bulb, natural wet-bulb, and black globe) into a single value. The WBGT index directly and indirectly measures key parameters of the ther-mal environment (i.e., temperature, humidity, wind, and radiation), and as a result provides a more comprehensive understanding heat stress risk than air temperature alone. Subsequently, it has been widely adopted in the categorisation of heat stress risk in sporting, military, and occupational contexts with adjustments for clothing level, work intensity and duration [12,13].

The ready-made garment (RMG) industry in Bangladesh is already impacted by heat, with WBGT frequently exceeding recommended thresholds for safe work practices [14,15]. The sector is key to Bangladesh’s economic and social development, contributing 12–15% of gross domestic product and 82% of the country’s exports by value [16,17]. It is a major employer to over 4 million people, of which 60% are estimated to be female [18]. Working conditions inside RMG factories are often difficult. Heat is one of many modifiable risk factors workers face day-to-day including, high particulate matter, excess noise levels, insufficient lighting, bullying, gendered roles, and high quotas that demand extended overtime work hours [19,20]. Research into improving worker health and wellbeing is required to improve the overall viability and productivity of the industry [16].

There is an urgent need to better prepare RMG workplaces via the provision of heat risk mitigation strategies, as conditions are only likely to worsen due to climate change. It is estimated that Bangladesh temperatures will increase by 3–4 °C by 2100 under a high emissions scenario [21,22] with temperatures in highly urbanised areas - where most of Bangladesh’s factories are located – possibly rising by an additional 4 °C [23]. Increases in temperature of this magnitude would come at great human and economic cost, given that just a 2 °C increase in temperatures is projected to see Bangladesh’s manual labour workforces lose 573 h of produc-tivity/person/year, more than doubling from 254 h/person/year currently [24]. Heat-related problems are already apparent: between 2009 and 2018 Bangladesh ranked seventh out of 219 worldwide population-weighted countries with respect to the most cooling degree days (i.e., mean daily temperatures > 18.3 °C where expensive and carbon-intensive cooling of ambient temperatures is recommended), with Dhaka ranking third out of all global cities [25].

Of the RMG factories that aim to combat heat stress, many typically do so by using natural ventilation, mechanical ventilation (e.g., exhaust and/or ceiling fans), evaporative cooling and, more rarely, air-conditioning [15]. In 2020, space cooling contributed almost 16% of global building electricity consumption [26]. By 2050, global reliance on energy-intensive air conditioning is projected to more than triple compared to 2017 [27] as developing economies mature and living standards rise [25]. Compounding the issue further is the leakage of potent halogenated refrigerants that contribute over one third of all greenhouse gases emitted from air-conditioner use [28]. If Bangladesh’s RMG industry comes to rely predominantly on mechanical air-conditioning to cool factory environments this will be maladaptive by increasing operational costs and adding to greenhouse gas emissions, and placing further pressure on existing energy supply infrastructure. [29,30]. More passive cooling strategies are needed which will be more sustainable in the long term.

Research on building adaptation strategies is vast. However, the efficiency of these strategies in improving industrial conditions in developing countries under the threat of climate change needs further investigation. Passive building adaptations are typically centred around adjusting the building envelope through the use of shading, natural ventilation, insulation, greening, and/or albedo modifications [31]. The roof is a key element of the building envelope. It is exposed to direct solar radiation and significantly contributes to the total cooling load of the building [31] particularly for low-rise factory buildings with large floor areas. Typically, Bangladesh RMG factories are constructed as multi-level low-rise reinforced cement concrete structures with brick walls, surrounded by other comparable buildings in high density industrial districts. Applying building adaptations through retrofitting the roof offers a potentially highly effective strategy to help reduce solar heat gains, and therefore occupational heat stress in the adjacent factory floor. Prior to constructing costly and potentially ineffective retrofits, modelling performance of viable options is an important first step to help inform the decision-making process for heat mitigation at the building level.

Here, we evaluate the effectiveness of four feasible, sustainable, passive rooftop retrofits in reducing heat exposures during factory operational hours, by minimising the radiative heating loads experienced through the RMG factory rooftop. We modelled a typical, mid-sized, non-air-conditioned RMG factory in Bangladesh and simulated the impact of these retrofits on present and future indoor climate conditions. The results presented here have implications for factory planning and retrofit design, and may inform policies that target worker health, well-being, and productivity.

## 2 Methodology

**Fig. 1:**
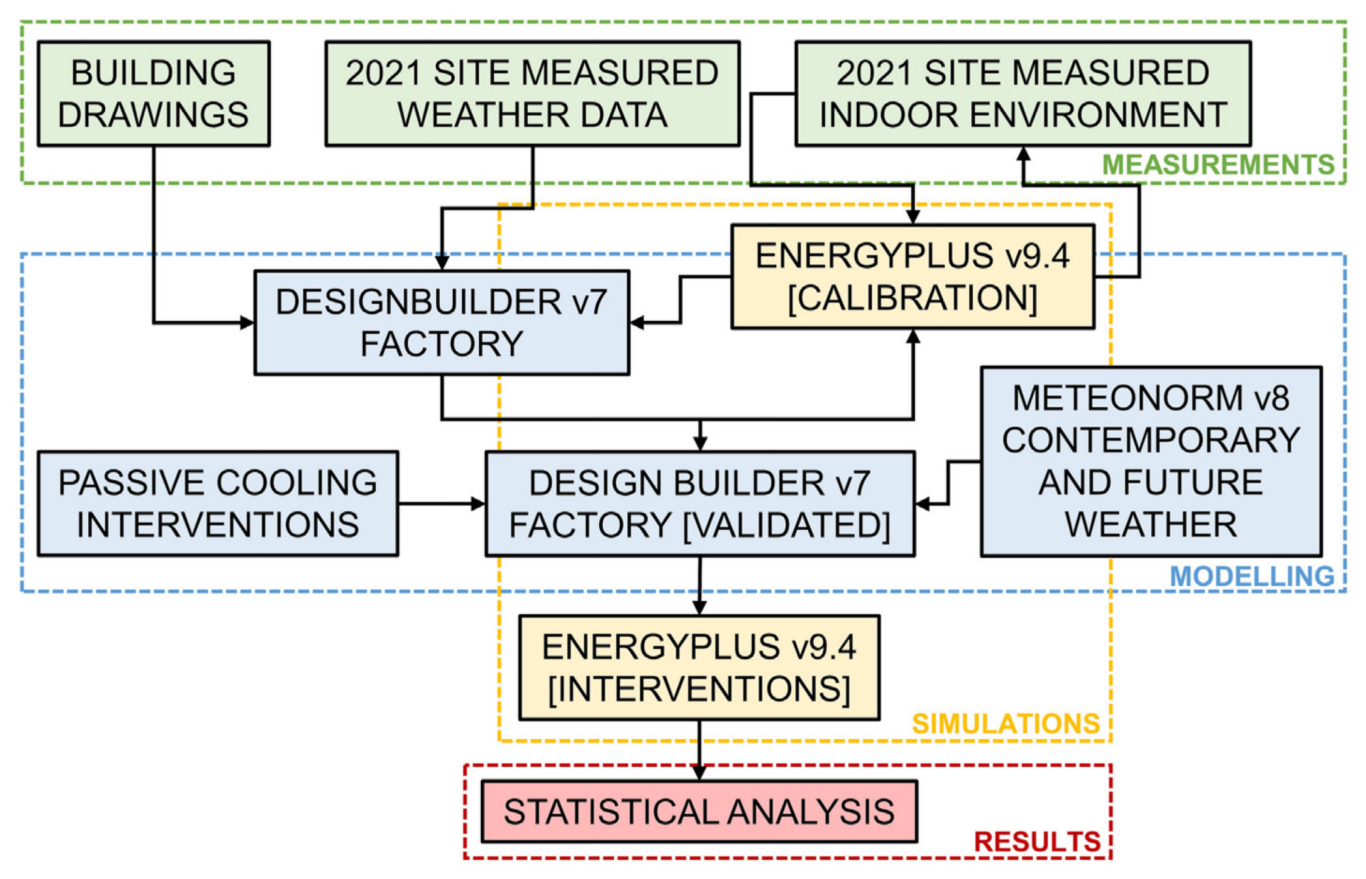
Schematic of study methodology.

The study methodology consisted of four distinct phases (Fig. 1). The first was *measurements*, whereby a physical RMG factory in Dhaka (Bangladesh) was extensively fitted with ambient monitoring sensors for the entirety of 2021. The second phase was *modelling*, consisting of the virtual construction of the factory, selection and virtual construction of possible passive cooling strategies at the building-level, and sourcing and selection of present climate and future climate weather files. The third phase was *simulations*, in which the calibration and validation of the virtual factory model based on the 2021 measured data took place. In addition, subsequent simulations were run of the combinations of building-level cooling strategies and various climate scenarios projected for future decades. In the final phase, *results*, collation and statistical analysis of all outputs took place to understand the performance of cooling strategies in the context of reducing occupational heat stress now and into the future.

### 2.1 Phase 1: Measurements

The typical, mid-sized, non-air-conditioned RMG factory produces clothing through assembly lines consisting of fabric cutting, sewing, ironing, quality checking, and packing. The factory has three storeys and is predominantly open plan, deploying extractor and ceiling fans on each floor to manage air circulation and remove air particulates, with some offices, toilets, and storage spaces located on the perimeter of each floor. The characteristics of the factory are outlined in Table 1.

#### Note

More detailed information of the factory, and parameters that informed the models that were taken from architecture plans, site surveys, and consultation with factory management are presented in the supplementary material (Table 1, Table 2).

**Fig. 2:**
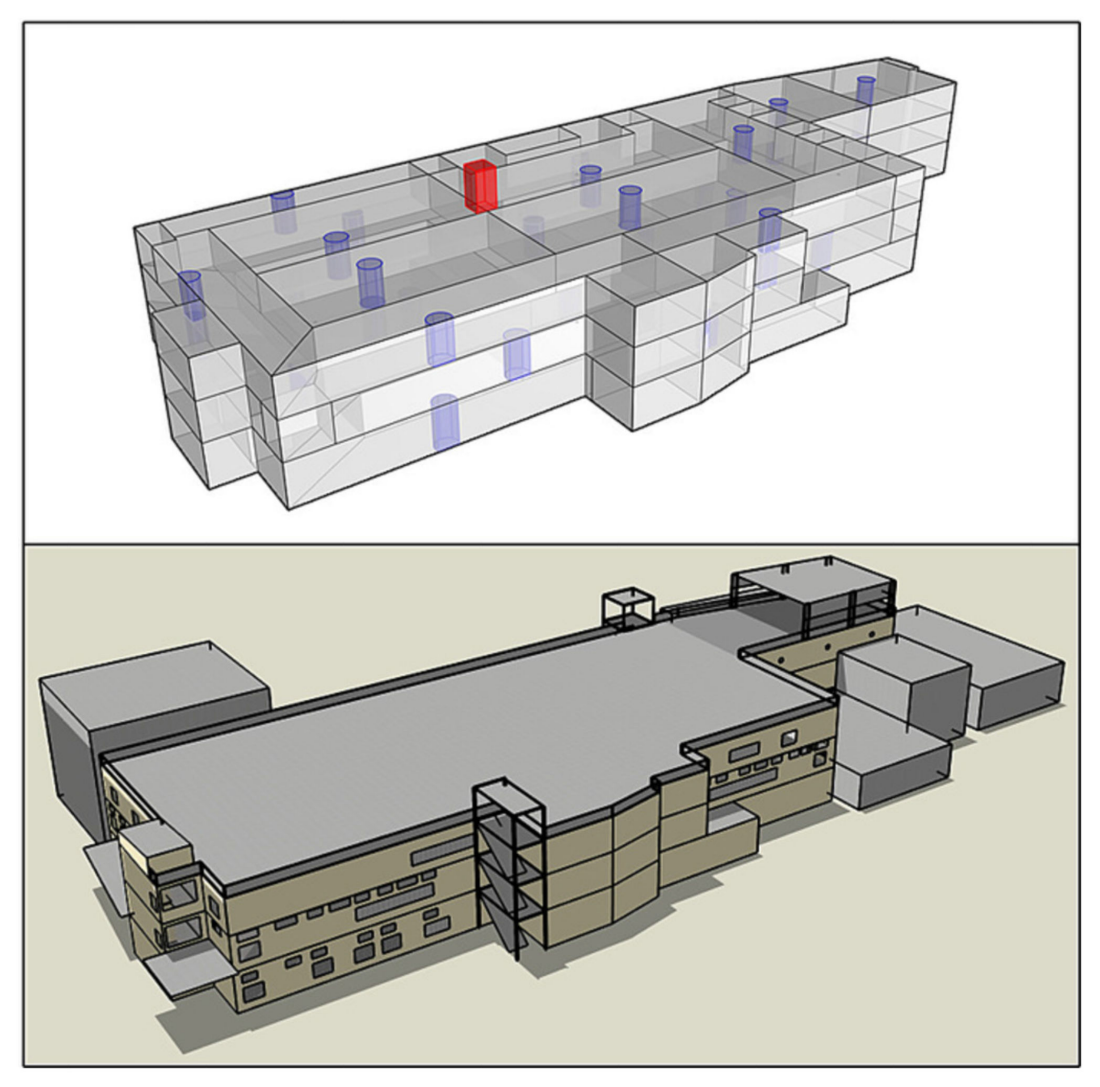
Virtual model of factory. a) rooms, and factory floor modelled zones, and position of environmental sensors [blue = indoor sensors, red = outdoor weather station], b) fully rendered factory model. (For interpretation of the references to colour in this figure legend, the reader is referred to the web version of this article.)

The factory was outfitted with internet of things (IoT) ambient sensors to collect internal and external environmental conditions for hot-spot identification and model validation. One meteorological weather station (Table 2) was installed on the factory rooftop (sensors 2.1 m above rooftop), and 33 temperature-humidity sensors (Table 2) were distributed across the three factory floors (Fig. 2). Indoor sensor placement was prioritised to the factory floor areas where workers were most often present and placed in the middle of the modelled perimeter and central zones measured from architectural plans (Fig. 2) [32]. Zones were defined by physical walls and modelled air-walls for zone boundaries, with physical sensors placed in the centre of zones at heights ranging 1.6 to 2.1 m above the floor to avoid artificial sources of heating/cooling and being lost/damaged by typical factory activities. Measurements were taken from January 1st until December 31st, 2021.

### 2.2 Phase 2: Modelling

DesignBuilder [33] (version 7.01.006) was used as the front-end interface for modelling the factory constructions (including building-level cooling strategies), occupancy and equipment schedules, as well as the heat loads from lighting, fans, and machinery (e.g., sewing machines, irons) (see supplement, Table 1). The dynamic thermal simulation was calculated by the underlying backend software EnergyPlus [34] (version 9.4.0).

For calibration and validation purposes, a weather file for the year 2021 was constructed using a combination of hourly external weather station data collected onsite (dry-bulb temperature, relative humidity, wind speed, wind direction), and parameters derived from satellite weather sources SOLCAST [35] (atmospheric pressure, global horizontal radiation, direct normal radiation, diffuse horizontal radiation), and the NASA Power Project [36] (total sky cover) taken from the nearest grid point from the factory.

To minimise the error between the predicted and measured hourly indoor temperatures, an EnergyPlus model of the factory was parametrically calibrated by aligning the top floor model output zone temperatures with those measured inside the factory top floor during a 4-day summer holiday period in 2021 when the factory was not operational. This process took the form of a genetic algorithm. Genetic algorithms have been successfully applied in calibrating energy models [37,38]. They are a subclass of evolutionary algorithms, which iteratively modify parameters within a solution space to minimise a dependent error function [39]. We specified construction parameters within an expected range for various physical properties such as solar absorptance, density, thickness, specific heat capacity, and thermal conductivity of the factory walls, roof, floors, and internal mass [40]. The algorithm fitness function minimised the sum of the squares of the distance between the zone interior air temperature curves at each time step, for each zone. The iteration repeats until the desired agreement between the measured and modelled conditions are met. Paramet-ric EnergyPlus model creation and split-core simulation was achieved with the python package EPPY [41], while automated data analysis was achieved with PANDAS [42]. Computational challenges were addressed by running the script in a virtual machine on the Australian Research Data Commons (ARDC) Nectar Research Cloud.

Following the 4-day unoccupied calibration, typical workday scheduling of lighting, equipment (e.g., sewing machines, irons, fans), and occupancy were identified. This was done through observation, discussion with facilities management of the factory and cross-checking with worker time-sheets. These workday schedules along with holidays were then incorporated into the factory model for the entire calendar year. To evaluate the accuracy of the final model over the entire year, we applied the methodology outlined in 4.2.11.1 of the ASHRAE Guideline 14-2014 [43] using the coefficient of variation of the root-mean-square error (CV [RMSE]), and normalised mean bias error (NMBE) (supplement, Equations 1 & 2). Comparisons were made across all hours of 2021, and the three seasons (cool-dry [Jan, Feb, Nov, Dec], hot-dry [Mar, Apr, May], hot-wet [Jun, Jul, Aug, Sept, Oct]), for each of the top floor zones in which workers were located. The ASHRAE Standard 90.1-2019 [43] considers CV(RMSE) and NMBE for hourly energy consumption calibration acceptable if modelled and measured data fall within ±30% and ±10%, respectively. These acceptable limits have been suggested to be too large for calibration based on indoor temperatures [38] which for similar research ranged from ±5% to ±14% for CV(RMSE), and ±2% to ±6% for NMBE.

### 2.3 Phase 3: Simulations

**Fig. 3:**
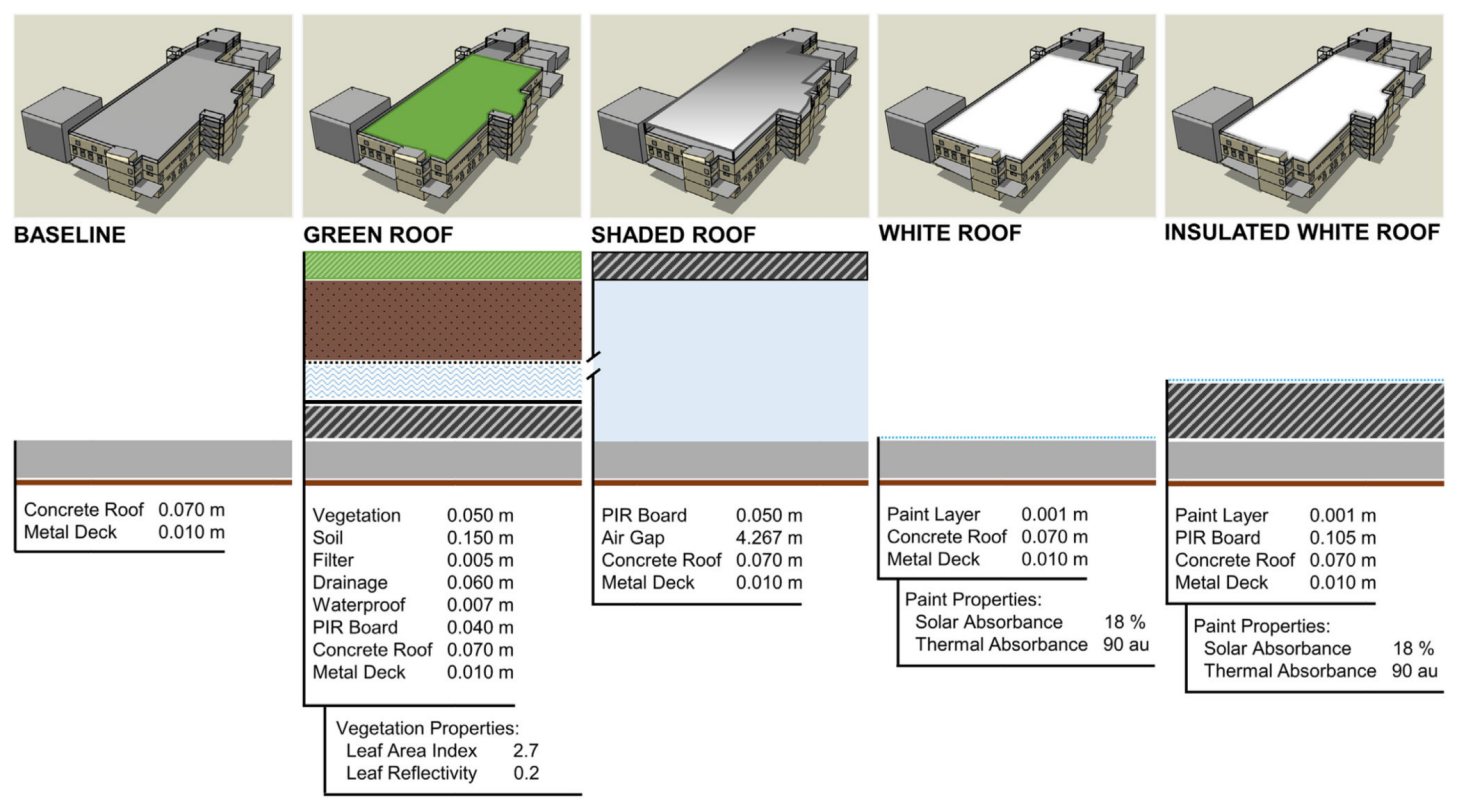
Constructions of simulated passive cooling strategies (PIR = Polyisocyanurate).

Along with the baseline factory model, Design Builder was used to model four feasible, passive cooling strategies that could be retrofitted to the existing factory roof: 1) an extensive green roof, 2) a shaded roof, 3) a white (cool) roof, and 4) an insulated white (cool) roof (Fig. 3). It is reasonable and realistic to simulate roof modifications for this building, since it was designed for future factory expansion of additional floors if required. Passive cooling strategies were selected from feasible options based on considerations of upfront and ongoing costs, maintenance, and practicality.

For the performance simulations, contemporary and future climate scenario weather files were generated through Meteonorm (version 8.0.3.15910). Meteonorm produces 10th, 50th and 90th percentile (P10, P50, P90) future weather files from a range of climate scenarios derived from ten global climate models from the CMIP5 ensemble (the Coupled Model Intercomparison Project Phase 5) [44,45]. The software applies a *‘morphing’* technique similar to other popular methods [46] through an autoregressive model that generates monthly time series data for key Intergovernmental Panel on Climate Change (IPCC) representative concentration pathway (RCP) scenarios [47]. For our baseline present-day weather file, we used the Meteonorm *Contemporary* (2000–2019) weather file interpolated from ground-measured and satellite data for the factory’s location. From this present-day file, future weather files were generated and selected for each decade for the expected life of the building: 2030 (P50), 2040 (P50) and 2050 (P50) across RCP2.6, RCP4.5 and RCP 8.5. Simulations for factory cooling strategies by climate scenario (i.e., contemporary, and future decades) were run on a virtual machine via the ARDC Nectar Research Cloud.

### 2.4 Phase 4: Analysis

The primary aim of the study was to determine the extent to which the passive cooling strategies reducing could reduce indoor heat stress, focusing on the areas of the factory where workers were at greatest risk of heat stress. The hottest area of the factory was determined using mean daily indoor dry-bulb temperature as measured during factory operation hours throughout 2021. Data were analysed using a generalised additive model, with factory level (floors: ground, middle, top) and workday (293 days) included as fixed effects, and a smooth term specified for workday. The model was implemented using the R package *mgcv* [48].

Odds ratios were used as a measure of association between a given roof type and a threshold limit WBGT temperature for both the present-day and for future decades. The equations used to derive WBGT from EnergyPlus outputs are presented in the supplement (Equations 3 and 4). Two separate logistic regression models were fitted, using hourly WBGT cut points of 28 °C and 30 °C, respectively, to determine the reductions in occupational heat risk thresholds relative to the existing roof. Each model included roof type, decade, and roof type by decade as fixed factors. WBGT thresholds of 28 °C and 30 °C represent the limits by which acclimatised workers at moderate (~300 W) and light (~180 W) metabolic rates can maintain a body core temperature < 38 °C throughout an 8 h working day [12]. The selected work rates correspond with the typical tasks of workers within the RMG factory (e.g., sewing, ironing, cutting, packing). As self-preservation measure against overheating, workers may modulate their own work intensity during periods of heat stress [49]. The nature of productivity-based employment in combination with hot working conditions can result in competing motivations. Should a worker downregulate their pace of work, they are faced with less take home pay or working longer hours in hot conditions to maintain the desired output. Keeping environmental exposures within the desired work rate specific WBGT thresholds will enable the workplace to maintain both worker health and productivity.

Results were reported as odds ratios and 95% confidence intervals. Analyses were undertaken in R [50] (version 4.2.1), using the RStudio [51] (version 2022.07.1 Build 554) environment. Data visualisations presented in this manuscript were produced using the R package *ggplot2* [52].

## 3 Results

**Fig. 4:**
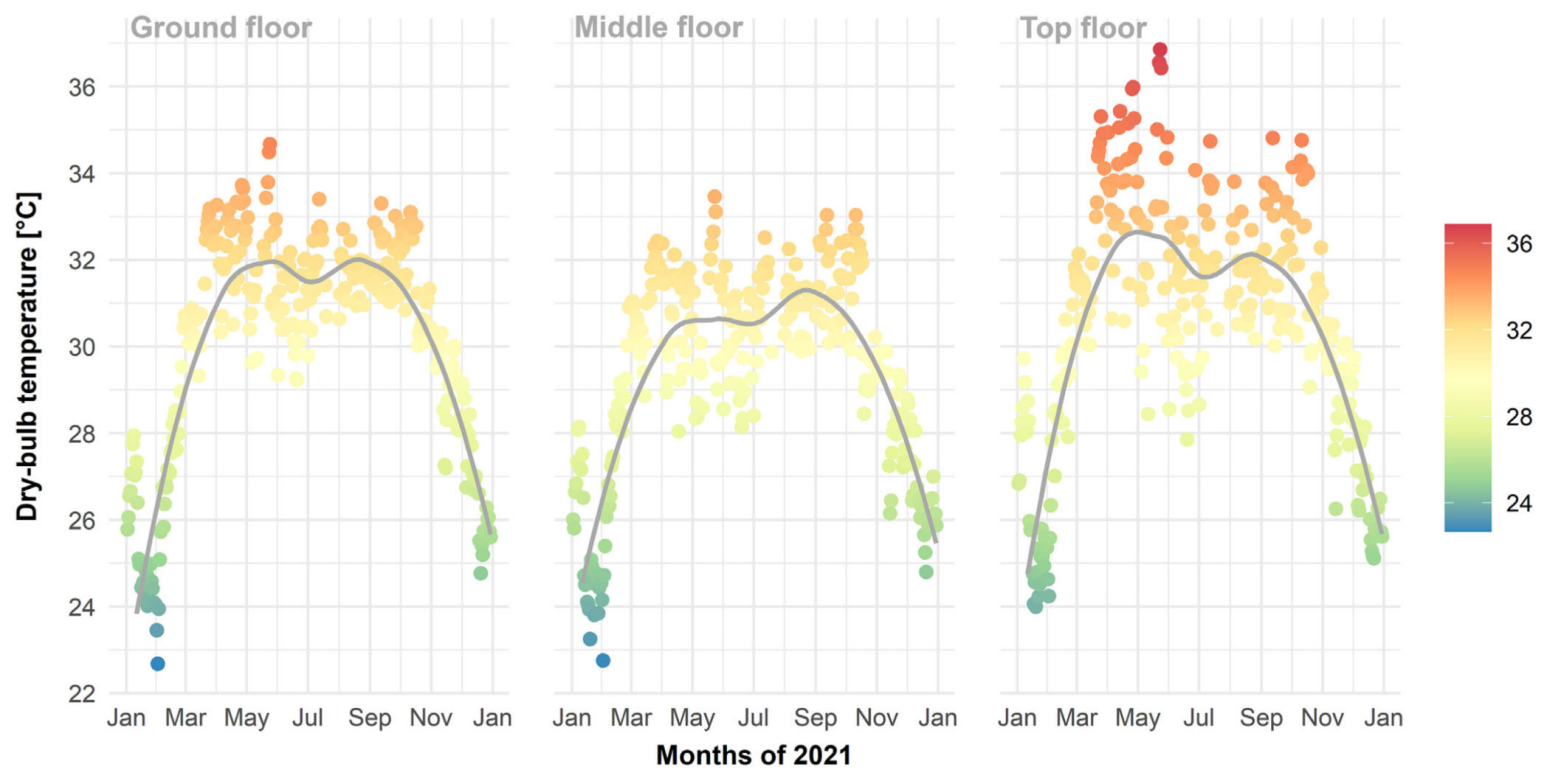
Measured mean daily indoor air temperature for each factory level on workdays in 2021.

The measured mean daily indoor dry bulb temperature was lower on the factory middle level than the ground floor (*β* = – 0.65, 95% CI = –0.85, –0.44; *p* <.001); and higher on the factory middle level than both ground (*β* = 0.50, 95% CI = 0.30, 0.71; *p* <.001) and top floors (*β* = 1.15, 95% CI = 0.94, 1.36; *p* <.001) (Fig. 4). The results of the top floor exclusively are now reported due to the observed need for reducing heat exposure on the top floor, combined with the minor impact rooftop interventions have on the indoor temperatures for the lower floors in a mechanical/-naturally ventilated factory.

The calibration of the factory model constructions by comparing modelled and measured temperatures during the 4-day unoc-cupied period is visualised in the supplementary material (Fig. 1). The final model parameters and construction properties are presented in the supplementary material (Table 1 and Table 2). The overall model agreement (NMBE and CV(RMSE)) between measured dry-bulb temperature was consistently within the respective ±10% and ±30% threshold limits [43] and within the narrower limits reported in the literature [38] across entire year (Table 3) and three seasons (supplement, Table 3).

Compared to the existing roof type, the four simulated roof types reduced the number of days workers experienced average daily conditions in excess of WBGT 28 °C and 30 °C, respectively (Table 4). The hourly and day counts for roof type performance over future decades and RCP climate scenarios 2.6, 4.5 and 8.5 are provided in the supplementary material (Table 4, Table 5, and Table 6). Relative to the existing roof, all simulated roof types, across all time points, saw significant reductions in odds for exposures above the WBGT thresholds (Table 5). However, there were no meaningful differences between any of the passive cooling strategies, across any decade or WBGT threshold.

**Fig. 5:**
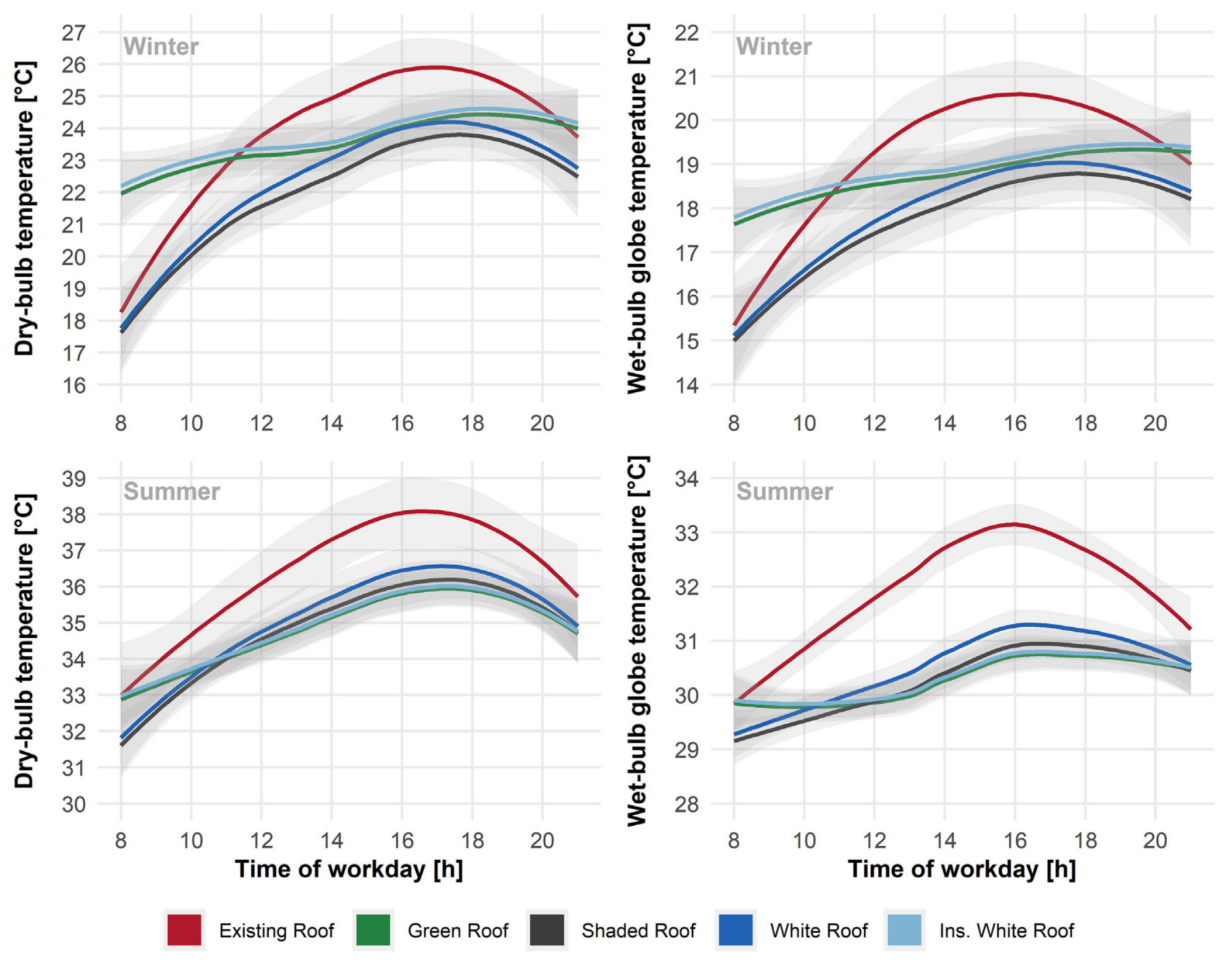
Present-day mean hourly indoor dry bulb temperatures and wet-bulb globe temperatures averaged over three consecutive cold winter and hot summer workdays. Data presented as mean (solid line) ±95% confidence intervals (grey shade).

**Fig. 6:**
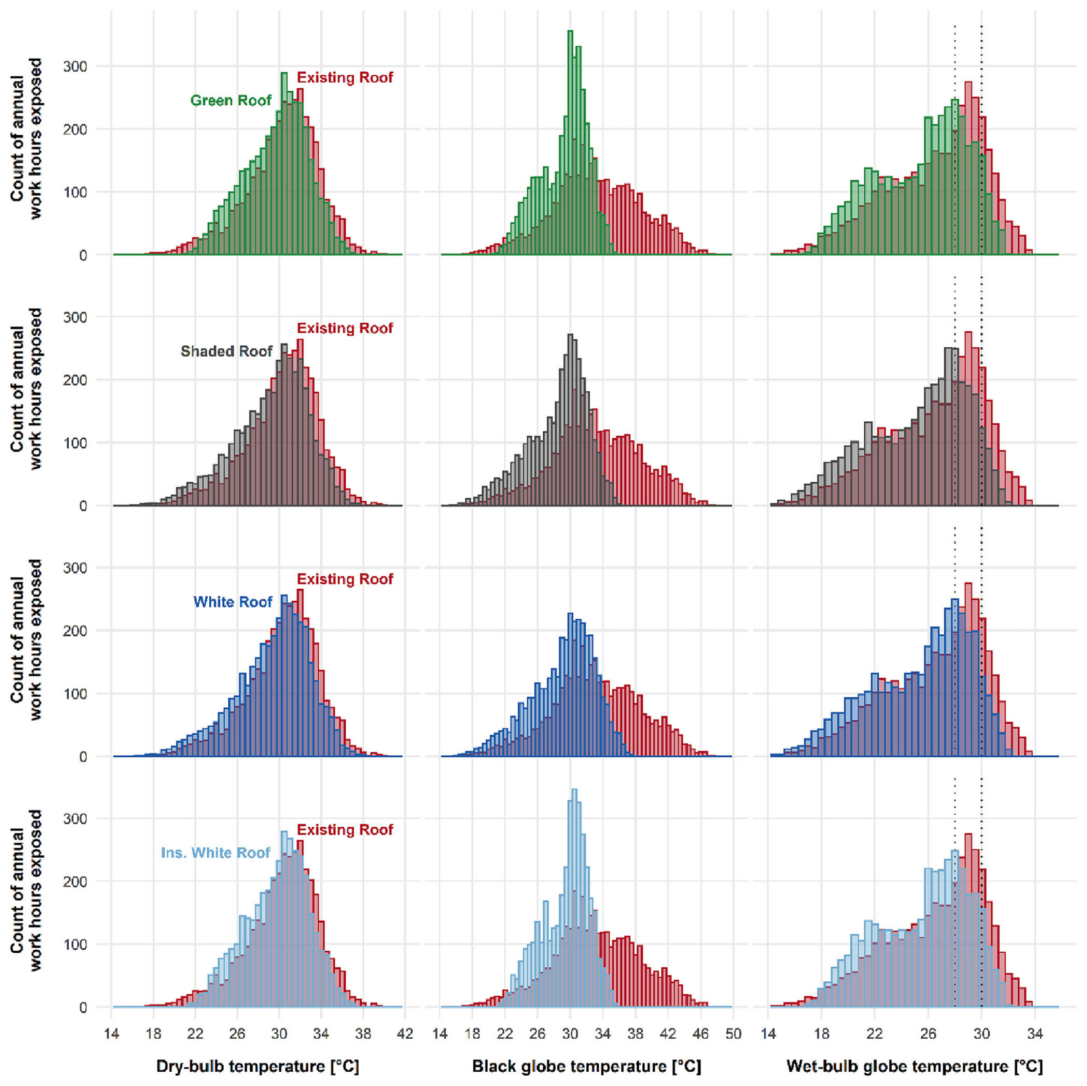
Present-day annual work hour distributions in indoor dry-bulb, black globe, and wet-bulb globe temperatures (WBGT) for different passive cooling strategies. WBGT thresholds of 28 °C and 30 °C (black dotted lines). Future decades under RCP 8.5 scenario displayed in supplement.

The relative performance of roof types on hourly profile for three consecutive workdays in both winter and summer is presented in Fig. 5. Regardless of the roof type, the biggest effect of employing passive cooling strategies for the roof comes from a reduction in radiative heat transfer as evidence by reductions in indoor black globe temperatures. This is illustrated in histograms of hourly counts across present-day annual factory operating hours for dry-bulb temperature, black globe temperature, and WBGT relative to the existing roof (Fig. 6). The green roof and white insulative roof had positive effects on reducing the extremes of both tails of the hourly indoor temperature distributions (Fig. 6). We observed higher minimum indoor temperatures during throughout the year (favourable in winter, unfavourable in summer) due to the insulating effect of these roof types (Fig. 5, Fig. 6). Future RCP8.5 decades of annual work hour temperature distributions are displayed in the supplementary material (Fig. 2, Fig. 3, and Fig. 4).

**Fig. 7:**
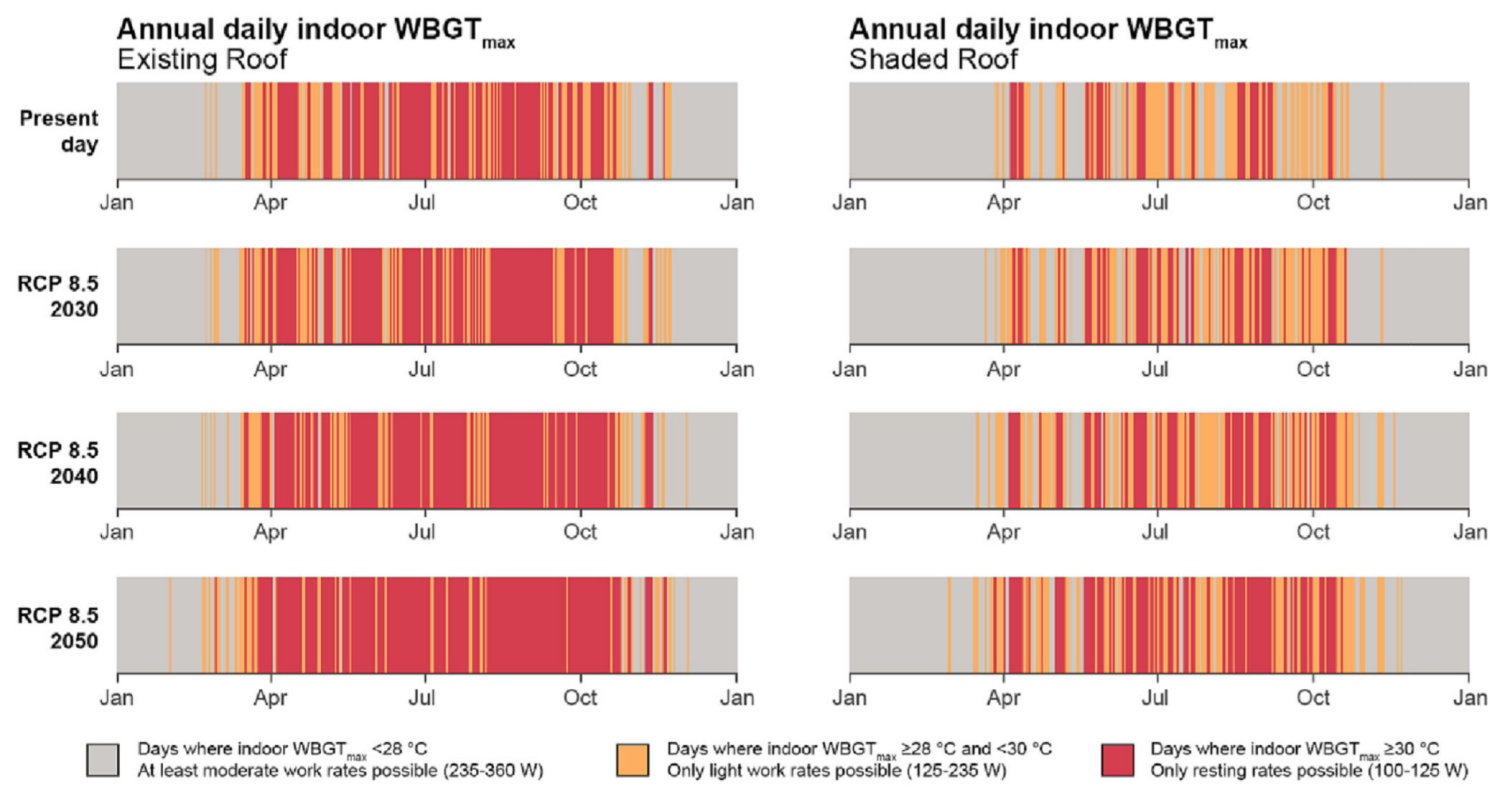
Annualised present-day and future decades (RCP 8.5) where daily indoor maximum wet-bulb globe temperatures (WBGT_max_) exceed work rate thresholds for at least 1 h; existing roof (left) and shaded roof (right). Work rate thresholds based upon ISO7243-2017 [12] for acclimatised workers.

**Fig. 8:**
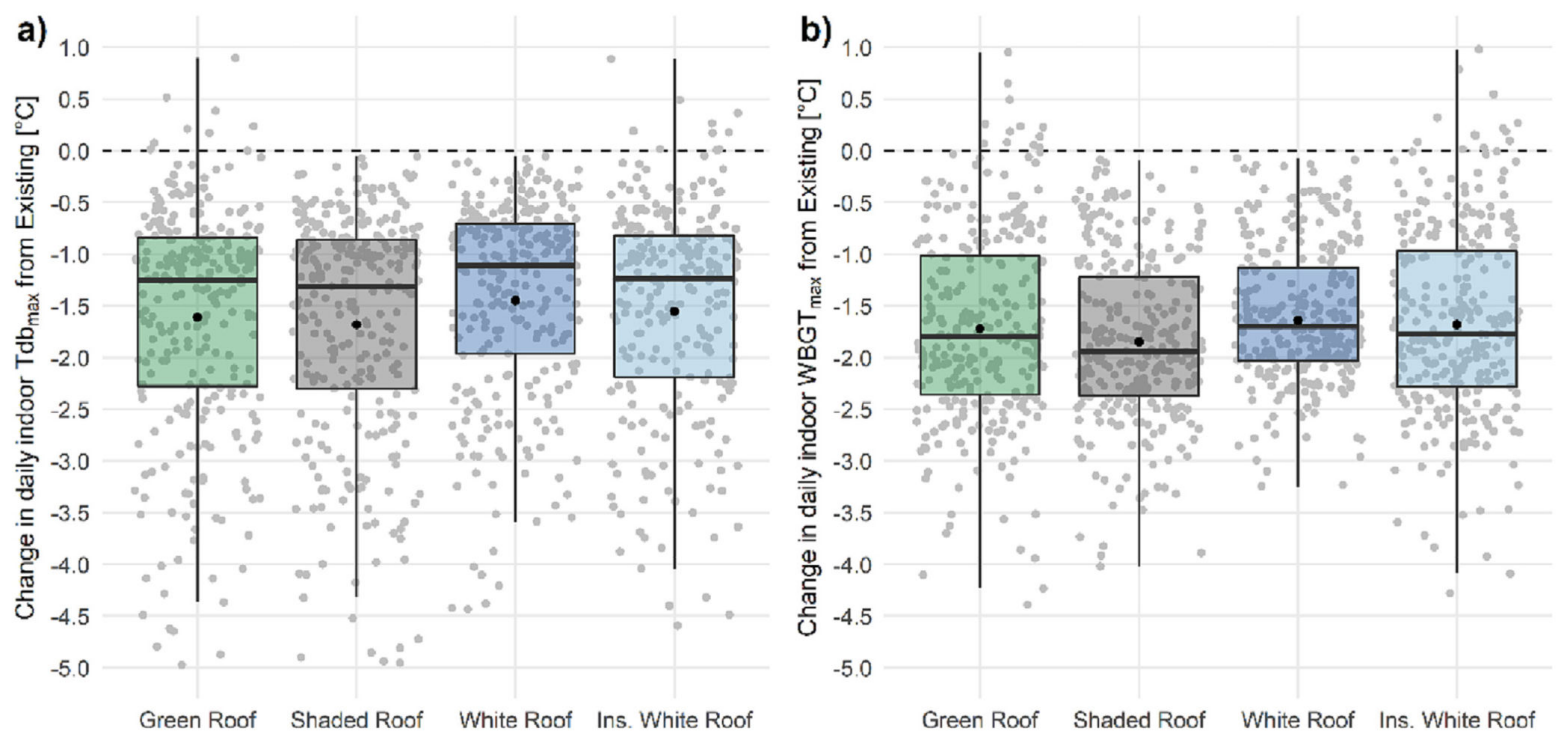
Present-day cooling roof types compared to existing roof for changes in indoor a) maximum dry-bulb temperature max (T_db_max_), and b) maximum wet-bulb globe temperature (WBGTmax) of annual workdays. Mean (solid circle), median (solid line).

For visualisation purposes, Fig. 7 presents annual days exceeding relevant work rate thresholds for the modelled daily indoor WBGTmax between the existing roof and shaded roof conditions across present-day and future decades under RCP 8.5. The annual differences between the control (no intervention) and the cooling strategies are, for indoor dry-bulb temperature across the top floor were −1.9 °C [95% CI: −2.1, −1.7 °C] for the green roof, −2.0 °C [2.2, −1.8 °C] for the shaded roof, −1.7 °C [-1.8, −1.5 °C] for the white roof, and −1.9 °C [-2.1, −1.7 °C] for the insulated white roof, (Fig. 8). The annual differences between the control and the cooling strategies for indoor WBGT_max_ were −1.7 °C [95% CI: −1.9, −1.6 °C] for the green roof, −1.9 °C [-2.0, −1.8 °C] for the shaded roof, 1.6 °C [−1.7, −1.6 °C] for the white roof, and −1.7 °C [−1.8, −1.6 °C] for insulated white roof (Fig. 8).

## 4 Discussion

The current study simulated the efficacy of retrofitting four passive rooftops of an RMG factory in Bangladesh as a means for reducing indoor heat exposure. All roof types significantly reduced the number of days and cumulative hours projected to exceed work rate specific WBGT thresholds. Improved projected working conditions with different rooftops were predominantly attributable to reduced radiative heating loads. All straggles significantly reduced the odds of exposure to work rate specific WBGT thresholds of 28 °C and 30 °C across present-day and future decades. Though, there were no significant differences in the odds between the different passive cooling strategies. The shaded and green roofs consistently provided the largest annual reductions in WBGT exceeding 28 °C and 30 °C by total work hours. For instance, present-day hours above WBGT 28 °C were reduced by 38% and 36% for the shaded and green roofs, respectively. While present-day hours above WBGT 30 °C were reduced by 62% and 56% for the shaded and green roofs, respectively.

To the best of our knowledge, no studies to date have compared the potential for a wide range of rooftop passive cooling strategies to reduce indoor heat exposures in RMG factory settings. Depending on the roof type, the number of present-day annual workdays and work hours on our factory top floor where indoor dry-bulb air temperatures exceed 35 °C were reduced by 22-29 days and 111-146 h compared to the existing roof. The number of annual workdays where indoor WBGT exceeded 30 °C was reduced by 55-61 days. Across the year, the simulated roofs reduced the daily maximum indoor air temperatures and WBGT by 1.7 to 2.0 °C and 1.6 to 1.8 °C, respectively. These findings are in line with other comparable studies. The use of white (cool) roofs applied in India [53] and Greece [54] to non-airconditioned buildings reported reductions in indoor air temperatures of between 1.5 and 2.0 °C, and 1.3 to 2.3 °C, respectively. A previous investigation of simulating green roof performance in Bangladesh RMG factories shows a reduction of indoor air temperatures of 2.5 to 3.5 °C [55]. Besides factory specific and modelling differences, these larger reductions found by Chowdhury et al. [54] as compared to the current investigation (1.7 °C annually) may be a result of their analysis only including the hottest months of April through July.

Passive cooling via rooftop interventions has the potential to reduce indoor temperatures [53], building energy consumption [56,57], and the urban heat island effect [58]. Our results indicate that, in a warming climate, the use of these rooftop cooling strategies can offset projected rises in indoor factory temperatures, with a comparable number of annual high heat exposure workhours and days in 2050 (RCP8.5) as presently experienced. Under a more optimistic climate change scenario such as RCP2.6, the number of days by 2050 exceeding these thresholds would be less than those presently experienced. The conditions currently faced by workers frequently exceed safe working standards as defined by developed economies. Ideally, minimising excessive heat in developing economies will result from scalable, economically viable, and sustainable cooling practices. Although the cooling effects of the passive strategies simulated in the current study do not minimise heat stress to the extent of carbon intensive air-conditioning, they offer an alternative, sustainable, and more environmentally friendly avenue for heat mitigation that can be incorporated into a wider range of solutions at the individual, building, and urban level [10,59].

The reduction of air temperature is not the only meaningful tool in preventing heat stress. One method of facilitating the release of excess heat from the body into the environment could simply be by increasing natural and/or forced convection to improve worker thermal comfort [15]. Under most ambient conditions, this will provide a net cooling effect by improving the efficiency of sweat evaporation [60]. Individual (e.g., fanning, dousing, clothing, hydration, work effort pacing), building (e.g., glazing, ventilation, coatings), and urban environment (e.g., infrastructure, water bodies, green spaces) modifications can all deliver cooling effects. Understanding how to combine these diverse strategies to achieve maximum impact is not straightforward, given the potential for synergistic and antagonistic interactions. Future translational research is warranted to better understand the impacts of multilevel combination cooling on worker health, wellbeing, and productivity during heat exposure.

Whilst the different strategies employed in the current investigation performed comparably, each has its own strengths and weaknesses depending on the applied context. Strategies range in their price, efficacy, feasibility, functionality, and ongoing maintenance requirements [10,61,62]. Shading of a building rooftop can be implemented in the form of low-cost, low-weight materials, insulated sheeting, and/or photovoltaic panels and, if desired, has the potential to still provide a usable space. Recent work has looked at the effect of city-wide simulations of different passive cooling strategies during hot weather [58,63]. At this scale, the albedo effect of the white (cool) roofs is the most effective at reducing city-wide temperatures and thus minimising the urban heat island effect [58]. Widespread adoption of green roofs may be advantageous beyond improving building conditions and energy consumption. For instance, green roofs can offer a recreational space, increase urban biodiversity, reduce air pollutants, and delay the quantity and improve the quality of rainwater runoff. [56,64]. However, the use of green roofs also has the downside of additional load bearing and structural requirements. Existing literature suggests the primary barriers to adoption of green roofs in Bangladesh are the absence of incentives from government to existing building owners, and the associated costs of ongoing maintenance [65]. Based on the current findings, insulated white (cool) roofs provide equivalent performance benefits as green roofs without the additional structural loads, though they do not provide functional usability or localised environmental benefits.

Our data indicates that the green roof and insulated white roof produce higher early morning temperatures during the beginning of work shifts, most likely due to the insulative effect that helps limit the heat discharged overnight from the buildings’ thermal mass (Fig. 5). This effect is advantageous in the cold winter periods, delivering warmer morning temperatures during months when conditions under the existing roof are unpleasantly cold (below 20 °C) at the start of shift. Despite white roofs being cheaper to implement and maintain than green roofs, economic analysis of their application in the United States across their 50-year life-cycle suggests the annualised cost premium from green roofs over white roofs are marginal [61]. More work is needed in determining life-cycle costs in the context of the Bangladesh RMG industry. Such an analysis should include not just local material costs, construction costs and potential energy savings but also the opportunity costs associated with reduced operational output due to heat-based reductions in workplace productivity via a lower physical work capacity [1,3] and higher absenteeism rates [5], as well as greater incidence of heat induced work-related accidents/injuries [4]. A significant number of Bangladesh’s RMG factory buildings are old and not purpose build for industrial activities, resulting in poor thermal condition. At the same time, major RMG trade partners and buyers are increasingly putting minimum requirements in place to ensure safe working conditions, mainly focusing on structural and fire safety but not so much on thermal condition. As a result, many of these factory buildings will require structural retrofitting within next 5 to 10 years to continue to meet major buyer requirements. This provides a good opportunity to include improvement of thermal condition as a part of these retrofits using sustainable cooling strategies as presented in this study.

There are several limitations to this study that should be considered when interpreting the results. First, it is important to keep in mind there are a variety of factors that determine the thermal performance of rooftop interventions, including the local climatic characteristics, whether they are a retrofit installation or new building design, building size and roof surface area, building orientation, building surroundings, building materials, and the specific characteristics of the rooftop intervention [66]. Despite our selected factory being representative of a typical, mid-sized, non-air-conditioned RMG factory, the simulation results of this study should not necessarily be assumed to hold true in other factories or different contexts. Second, during simulations the performance of the rooftops are static despite their application in the real-world showing degradation over time. For instance, finding low maintenance, resilient native flora for extensive green roofs is difficult and will impact performance [62]. White (cool) roofs see deterioration in performance over time with general weathering and accumulation of dirt that requires continual cleaning and recoating [67]. Third, the WBGT work rate specific thresholds applied in this study [12] assume a *‘best case scenario’* with healthy workers undertaking adequate hydration practices, who are acclimatised to heat and wear similar clothing. All these variables may shift the work-rate thresholds applied in this study to even lower WBGT values in order to be deemed safe.

## 5 Conclusion

The purpose of this study was to identify passive factory cooling strategies that would be effective now and, in the future, without adding to the burden of greenhouse gas emissions. We simulated four strategies: a green roof, rooftop shading, a white (cool) roof and an insulated white roof. The shaded roof saw the greatest reduction in both dry-bulb temperature and WBGT safe work thresholds, though all strategies were comparable in terms of their impact. We found that all evaluated strategies reduce indoor air temperatures by around 2 °C on average and reduce the number of present-day annual work-hours in which WBGT exceeds the safe limits for moderate work rates by up to 603 h - the equivalent of 75 (8 h) working days each year. All strategies simulated temperature reductions in future climate change scenarios; by 2050 factory conditions with a rooftop intervention are comparable to present-day conditions. The results of the study can help decision makers develop and implement sustainable cooling solutions to help improve working conditions while minimising greenhouse gas emissions within the Bangladesh RMG industry. Further work is required to understand the validity, feasibility, and economics of these strategies in the real-world and their performance in combination with other personal cooling strategies as viable alternatives to air-conditioning.

## Supplementary Material

Supplementary data to this article can be found online at https://doi.org/10.1016/j.enbuild.2023.112954.

Supplementary file

## Figures and Tables

**Table 1:** Building characteristics.

Build Year	2013		
Building Type	Reinforced cement concrete structure, brick walls, single glazed windows		
	Ground Floor	Middle Floor	Top Floor
Floor Area (m^2^)	2461	2435	2435
Production Space (m^2^)	1802	2249	1783
Office Space (m^2^)	77	37	452
Storage/Other (m^2^)	582	149	200
Ceiling Height (m)	3.95	3.52	3.52
Typical Occupancy	600	1000	450
Mixed-Mode	Nil	Evaporative	Nil
Operation		cooling pads	
Mechanical	Exhaust Fans	Exhaust Fans	Exhaust Fans
Ventilation			
Door/Window Operation	Limited (kept open)	Limited (kept open)	Limited (kept open)

**Table 2:** Environmental monitoring sensor specifications.

	Make/Model	Sample Rate	Resolution	Accuracy
Outdoor Weather Station				
Dry-bulb Temperature	Environdata/TA70	<1 sec	0.02 °C	± 0.1 °C
Black Globe Temperature	Environdata/BG70	(averaged/60 min)	0.025 °C	± 0.1 °C
Relative Humidity	Environdata/RH40		0.1 %	± 2.0 % (10-90 %) at 25 °C
Air Speed	Environdata/WS52		0.1 kmoh-^1^	± 2.0-3.0 %
Air Direction	Environdata/WD50		0.1°	± 1°
Indoor Ambient Sensors				
Dry-bulb Temperature	Wireless Tag/Pro	30 min	0.02 °C	± 0.3 °C (5-60 °C)
Relative Humidity		(averaged/60 min)	0.12 %	± 2.0 % (20-80 %) at 25 °C

**Table 3:** Annual agreement between model and measured indoor dry-bulb temperatures.

Zone	Measured [h]	Missing Data [%]	NMBE [%]	CV(RMSE) [%]
1	6,957	-21	-0.19	4.63
2	7,486	-15	0.35	4.82
3	7,800	-11	0.65	5.10
4	7,815	-11	1.75	5.85
5	6,908	-21	-0.82	4.78
6	6,055	-31	-1.68	5.17
7	6,462	-26	-0.75	4.55
8	7,554	-14	0.34	4.29
9	6,706	-23	-2.05	4.99
All	63,743	-19	-0.17	4.94

NMBE = normalised mean bias error, CV(RMSE) = coefficient of variation root mean square error. Acceptable NMBE for hourly data = ±10%. Acceptable CV(RMSE) for hourly data = ±30% [43].

**Table 4:** Number of working days (n = 293) and hours (n = 3,708) exceeding indoor dry-bulb temperature (T_db_) and wet-bulb globe temperature (WBGT) thresholds for present-day and future decades under RCP8.5.

	Existing		Green		Shaded		White		Ins. White	
	Days	Hours	Days	Hours	Days	Hours	Days	Hours	Days	Hours
**Tdb** ≥ **30** °**C**									
Present-day	244	2,188	***205***	1,907	***205***	***1,776***	208	1,845	206	1,930
RCP8.5 2030	248	2,246	208	1,985	***206***	***1,856***	211	1,905	209	2,007
RCP8.5 2040	257	2,481	224	2,239	***221***	***2,104***	231	2,176	227	2,265
RCP8.5 2050	268	2,684	233	2,436	***232***	***2,273***	239	2,339	236	2,460
**Tdb** ≥ **35** °**C**									
Present-day	51	227	***22***	***81***	23	90	29	116	23	92
RCP8.5 2030	72	351	***36***	***169***	39	174	43	201	36	172
RCP8.5 2040	99	519	***52***	**272**	57	274	65	311	52	278
RCP8.5 2050	108	587	***55***	323	61	***318***	70	368	56	333
**WBGT** ≥ **28** °**C**									
Present-day	176	1,602	***119***	1,031	***119***	***999***	130	1,088	***119***	1,038
RCP8.5 2030	188	1,747	138	1,304	***137***	***1,232***	143	1,320	140	1,317
RCP8.5 2040	188	1,903	***156***	1,521	157	***1,466***	164	1,557	158	1,545
RCP8.5 2050	199	2,070	168	1,748	***166***	***1,675***	171	1,735	170	1,760
**WBGT** ≥ **30** °**C**									
Present-day	99	621	44	274	***38***	***235***	44	274	44	277
RCP8.5 2030	119	838	55	320	***54***	***301***	65	368	56	326
RCP8.5 2040	136	1,048	***68***	494	70	***474***	83	556	68	500
RCP8.5 2050	154	1,258	***92***	720	***92***	***675***	105	762	***92***	730

***Bolded-italics*** = lowest counts for days/hours relative to the existing roof.

**Table 5:** Odds ratios for indoor wet-bulb globe temperature (WBGT) exceeding thresholds for light and moderate work in acclimatised workers across roof type and decades for RCP8.5.

	WBGT ≥ 28 °C – Moderate work rates (-300 W)			
	Present-day	2030	2040	2050
**Existing**	1	1	1	1
**Green**	0.79 (0.74–0.83)	0.82 (0.77–0.87)	0.83 (0.77–0.89)	0.84 (0.77–0.90)
**Shaded**	0.78 (0.74–0.82)	0.79 (0.74–0.84)	0.81 (0.75–0.86)	0.81 (0.75–0.87)
**White**	0.80 (0.76–0.85)	0.82 (0.77–0.88)	0.84 (0.78–0.90)	0.83 (0.77–0.90)
**Ins. White**	0.79 (0.75–0.84)	0.82 (0.77–0.87)	0.83 (0.78–0.90)	0.84 (0.78–0.91)
	**WBGT ≥ 30 °C – Light work rates** (-180 W)			
	Present-day	2030	2040	2050
**Existing**	1	1	1	1
**Green**	0.90 (0.87–0.93)	0.85 (0.82–0.88)	0.83 (0.80–0.86)	0.82 (0.78–0.86)
**Shaded**	0.89 (0.87–0.91)	0.84 (0.82–0.87)	0.82 (0.79–0.86)	0.81 (0.77–0.85)
**White**	0.90 (0.87–0.93)	0.86 (0.83–0.89)	0.84 (0.81–0.88)	0.83 (0.79–0.87)
**Ins. White**	0.90 (0.88–0.93)	0.85 (0.82–0.88)	0.83 (0.80–0.86)	0.82 (0.79–0.86)

Data reported as odds ratios (95% confidence intervals). Odds ratio < 1 represents lower odds of hourly exposure above specified WBGT threshold compared to Existing roof (i.e., a protective exposure). All interventions significantly less than the existing roof. No significant differences were observed between interventions. Work rate specific WBGT thresholds based on acclimatised workers from ISO7243-2017 [12].

## Data Availability

Data will be made available on request.
