# Supplementary material for: Retrofitting passive cooling strategies to combat heat stress in the face of climate change: A case study of a ready-made garment factory in Dhaka, Bangladesh
Source: Energy Build. Author manuscript; Available in PMC 2023 Aug 20. (PMC7614966; doi:10.1016/j.enbuild.2023.112954)
Supplement: Supplementary file [file EMS184998-supplement-Supplementary_file.docx]

**Supplement**

# *[Model Parameters](#Contructions)*

# [*Equations*](#Equations)

# [*Results*](#Results)

# [*References*](#References)

*1. Model Parameters*

**Table 1.** Final model parameters and characteristics.

| **Simulation Parameters** | **Description** |
| --- | --- |
| Geographical location | Dhaka, Bangladesh |
| Longitude | 90.38 |
| Latitude | 23.89 |
| Time zone | +6 GMT |
| Simulation period | January 1^st^ - December 31^st^ |
| Sky model | Clear sky |
| Units of dimensions | Metric (SI) |
| Elevation | 11 m |
| Orientation | Front elevation facing south |
| Total number of floors | 3 |
| Simulated ﬂoor | Top floor |
| Floor to floor height | Ground: 3.95 m, Middle: 3.525 m, Top: 3.525 m. (Including the thickness: 0.07 m) |
| Floor area | Total: 7,229 m^2^, Ground: 2,427 m^2^, Middle: 2,401 m^2^, Top: 2,401 m^2^. |
| Window area | 248 m^2^ windows (8.6% window-to-wall ratio) |
| Exterior solar absorbance | Walls: 0.8, Concrete roof: 0.90 |
| Occupancy density | Per the actual information: Ground: 0.272 people·m^-2^, Middle: 0.445 people·m^-2^, Top: 0.25 people·m^-2^ |
| Electric equipment | Per the actual information: Ground: 18.27 W·m^-2^, Middle: 53.53 W·m^-2^, Top: 20.36 W·m^-2^ |
| Glazing | Single glass clear: 6 mm, U-value: 5.8, Solar heat gain coefficient: 0.86, + modelled blinds |
| Natural ventilation | Calculated natural ventilation settings; calculates the airflows through fans and windows |
| Room air distribution model | Mixed air temperature distribution setting (default) |
| Ventilation control mode | Ventilation is scheduled with fans schedule |
| Design flow rate | Total of 47 factory exhaust fans. Max airflow: 485.83 m^3^·s^-1^. Part-load schedules are used to control them |
| Leakage component name | Crack template is used to model infiltration. |
| Heat balance algorithm | Conduction transfer function |
| Operation schedule | Compact schedule, based off factory worker time sheets for the 2021 year |
| Lights schedule | Custom schedule. Typical workday example: Until: 07:30, 0, Until: 13:00, 1, Until: 14:00, 0, Until: 21:00, 1, Until: 24:00, 0. |
| Equipment schedule | Custom schedule. Typical workday example: Until: 07:30, 0, Until: 07:50, 0.2, Until: 08:00, 0.9, Until: 13:00, 1, Until: 14:00, 0, Until: 21:00, 1, Until: 24:00, 0. |
| Occupancy schedule | Custom schedule. Typical workday example: Until: 07:30, 0, Until: 07:50, 0.2, Until: 08:00, 0.9, Until: 13:00, 1, Until: 14:00, 0, Until: 21:00, 1, Until: 24:00, 0. |
| Exhaust fan schedule | Custom schedule. Typical workday example: Until: 07:30, 0.05, Until: 07:55, 0.2, Until: 08:00, 0.6, Until: 13:00, 0.9, Until: 14:00, 0.1, Until: 19:00, 0.9, Until: 24:00, 0.05. |
| Inﬁltration schedule | Infiltration is always ‘on’. Calculated option is used - model simulates the cracks and openings |
| Clothing schedule | 0.74 clo (fixed) |
| Roof | 0.01 m metal deck + 0.09 m concrete + 0.01 m mortar and 0.01 m ceramic tiles where installed |
| Floor | 0.01 m metal deck + 0.07 cm concrete + 0.01 cm mortar + 0.01 cm ceramic tile |
| Wall | 0.01 m plaster + 0.15 m brick + 0.01 m plaster |
| Opening | Single glass clear: 6 mm, U-value: 5.8, Solar heat gain coefficient: 0.86, + modelled blinds |
| Shading | Modelled internal blinds, surrounding buildings, trees, etc. |
| Material speciﬁcation | According to BNBC and ANSI/ASHRAE Standard (R, Cp, S, k) |
| Output variable | Air Temperature (°C) |
| Time steps per hour | 4 |
| Ground temperature | Fixed at 24.5 °C |
| Output schedule | Hourly |
| Output option type | Simple and tabular, ESO formatted results are used |

**Table 2.** Modelled factory construction properties

| **Building Element** | **Total Area (m^2^)** | **Materials (layers)** | **Solar absorptance** | **Thermal conductivity (W·mK^-1^)** | **Density (kg·m^-3^)** | **Specific heat (j·kg^-1^K^-1^)** | **Total U-value (W·m^-2^K^-1^)** |
| --- | --- | --- | --- | --- | --- | --- | --- |
| External walls | 770.9 | 10 mm gypsum plastering | 0.5 | 0.43 | 2375 | 753 | 2.728 |
|  |  | 156 mm brick | 0.6 | 1.04 | 1025 | 750 |  |
|  |  | 10 mm gypsum internal plastering | 0.9 | 0.43 | 2375 | 753 |  |
| Internal walls | 1321.7 | 10 mm gypsum plasterboard | 0.5 | 0.25 | 900 | 1000 | 2.329 |
|  |  | 100 mm brick | 0.6 | 1.12 | 1764 | 1213 |  |
|  |  | 10 mm gypsum plasterboard | 0.5 | 0.25 | 900 | 1000 |  |
| Floor | 2435 | 10 mm ceramic tiles | 0.9 | 0.8 | 1700 | 850 | 2.534 |
|  |  | 10 mm mortar | 0.6 | 0.88 | 2800 | 896 |  |
|  |  | 135 mm concrete | 0.9 | 1.34 | 2487 | 753 |  |
| Roof | 2435 | 10 mm metal deck | 0.7 | 45.28 | 7824 | 500 | 5.209 |
|  |  | 70 mm concrete | 0.78 | 1.35 | 1400 | 600 |  |
|  |  | 10 mm metal deck | 0.7 | 45.28 | 7824 | 500 |  |
| Windows | 37 | 3mm clear glass | - | 0.9 | - | - | 5.894 |
|  |  | Aluminium frame | - | - | - | - |  |
| External wall exhaust fans | 17.9 | Galvanised steel frame and aluminium blades | - | - | - | - | - |

*2. Equations*

ASHRAE Guideline 14-2014 states that a model can be considered calibrated if NMBE < 5% and CV(RMSE) < 15% when monthly data are used, or NMBE <10% and CV(RMSE) <30% when hourly data are used. Because CV(RMSE) is the positive average sum-squared error divided by the actual mean, it can be considered the percent error between the simulation and measured data. Because NMBE is a signed error divided by the mean, it indicates bias percent for under- (NMBE > 0) or overshooting (NMBE < 0) the actual data during the period of evaluation.

$\text{NMBE = }\frac{\frac{\text{1}}{\text{N}}\sum_{\text{i}}^{\text{N}} \text{(}\text{y}_{\text{i}}\text{-}{\hat{\text{y}}}_{\text{i}}\text{) }}{\bar{\text{y}}}$ [1]

where,

NMBE = normalised mean bias error (%)

*N* = total number of data points

*y*_i_ = measured values

$\hat{\text{y}}$*_i_* = modelled values

$\bar{\text{y}}$*_i_* = average of measured values

$\text{CV}\left( \text{RMSE} \right)\text{ =} \frac{\sqrt{\frac{\text{1}}{\text{N}}\sum_{\text{i}}^{\text{N}} \text{(}\text{y}_{\text{i}}\text{-}{\hat{\text{y}}}_{\text{i}}\text{)}^{\text{2}}\text{ }}}{\bar{\text{y}}}$ [2]

where,

CV(RMSE) = coefficient of variation of the root mean squared error (%)

*N* = total number of data points

*y*_i_ = measured values

$\hat{\text{y}}$*_i_* = modelled values

$\bar{\text{y}}$*_i_* = average of measured values

Wet-bulb globe temperature (WBGT) as calculated using the outdoor equation – as radiant heat loads were so high indoors – variables were derived from the model outputs, dry-bulb temperature, relative humidity, and mean radiant temperature. First, wet-bulb temperature (*T*_wb_) was calculated from dry-bulb temperature (*T*_db_) and relative humidity using Equation 2. Mean radiant temperature was assumed to equal globe temperature (*T*_g_), then WBGT was calculated using Equation 3.

*T*_wb_ = *T*_db_ · *arctan*(*x*^1^(*ϕ* + *x*^2^)^1/2^) + *arctan*(*T*_db_ + *ϕ*) – *arctan*(*ϕ* – *x*^3^) + *x*^4^(*ϕ*)^3/2^ · *arctan*(*x*^5^ · *ϕ*) – *x^6^* [3]

where,

*T*_wb_ = wet-bulb temperature (°C)

*arctan* = invers of the tangent function

*c*_1_ = -8.78469475556

*c*_2_ = 1.61139411

*c*_3_ = 2.33854883889

*c*_4_ = -0.14611605

*c*_5_ = -0.012308094

*c*_6_ = -0.0164248277778

*T*_db_ = dry-bulb temperature (°C)

*ϕ* = relative humidity (%)

*WBGT* = 0.1·*T*_db_ + 0.2·*T*_g_ + 0.7·*T*_wb_ [4]

where,

*WBGT* = wet-bulb globe temperature (°C)

*T*_db_ = dry-bulb temperature (°C)

*T*_g_ =globe temperature (°C)

*T*_wb_ = wet-bulb temperature (°C)

*3. Results*


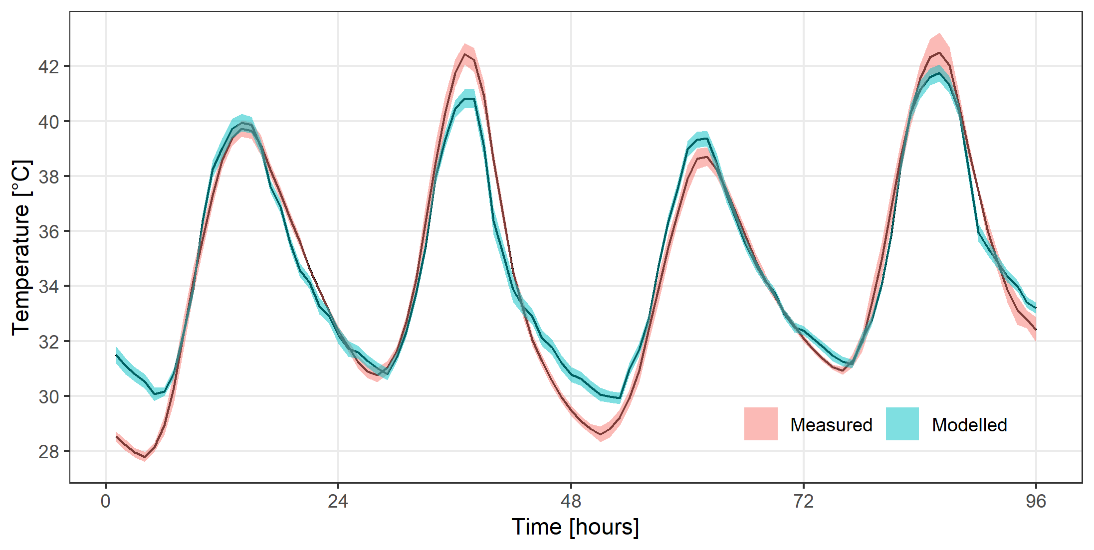


**Figure 1.** Visualisation of model construction calibration

Mean (line) and standard deviation (shade) of the *measured* zones and *modelled* zones during 4-day factory holiday closure period

**Table 3.** Seasonal agreement between model and measured dry-bulb temperatures

|  | **Cool-Dry Season** | | | | **Hot-Dry Season** | | | | **Hot-Wet Season** | | | |
| --- | --- | --- | --- | --- | --- | --- | --- | --- | --- | --- | --- | --- |
| Zone | Measured (h) | Missing Data (%) | NMBE | CV(RMSE) | Measured (h) | Missing Data (%) | NMBE | CV(RMSE) | Measured (h) | Missing Data (%) | NMBE | CV(RMSE) |
| 1 | 2,024 | -30 | -0.46 | 5.57 | 1,979 | -10 | -0.30 | 5.12 | 2,954 | -20 | 0.05 | 3.57 |
| 2 | 2,560 | -11 | 0.27 | 5.72 | 2,054 | -7 | -0.50 | 5.13 | 2,872 | -22 | 1.03 | 3.77 |
| 3 | 2,616 | -9 | 1.91 | 6.35 | 2,126 | -4 | -0.27 | 5.26 | 3,058 | -17 | 0.37 | 3.85 |
| 4 | 2,439 | -15 | 3.15 | 7.18 | 2,006 | -9 | 1.40 | 5.78 | 3,370 | -8 | 1.08 | 4.95 |
| 5 | 1,955 | -32 | -1.55 | 6.28 | 1,774 | -20 | -0.92 | 5.10 | 3,179 | -13 | -0.38 | 3.63 |
| 6 | 1,649 | -43 | -2.88 | 6.01 | 1,652 | -25 | -0.39 | 5.79 | 2,754 | -25 | -1.90 | 4.21 |
| 7 | 2,246 | -22 | -1.29 | 5.17 | 1,595 | -28 | 0.05 | 4.86 | 2,621 | -29 | -0.87 | 3.83 |
| 8 | 2,106 | -27 | 0.20 | 4.88 | 2,052 | -7 | 0.22 | 4.55 | 3,396 | -8 | 0.51 | 3.77 |
| 9 | 2,336 | -19 | -4.72 | 6.14 | 1,744 | -21 | -1.69 | 5.15 | 2,626 | -28 | -1.28 | 3.87 |
| **ALL** | **19,931** | **-23** | **-0.25** | **6.00** | **16,982** | **-15** | **-0.23** | **5.21** | **26,830** | **-19** | **-0.08** | **3.98** |

**Table 4.** Effect of passive cooling strategies relative to no intervention (Existing) on maximum indoor dry-bulb (*T*_db_max_), and wet-bulb globe temperatures (*WBGT*_max_) for annual working days (*n* = 293) for present-day and future decades under RCP2.6.

|  | **Existing** | **Green Roof** | | | **Shaded Roof** | | | **White Roof** | | | **Insulated White Roof** | | |
| --- | --- | --- | --- | --- | --- | --- | --- | --- | --- | --- | --- | --- | --- |
|  | # days | # days | Δ days | Δ % | # days | Δ days | Δ % | # days | Δ days | Δ % | # days | Δ days | Δ % |
| **Days *T*_db_max_ ≥ 30 °C** | | | | | | | | | | | | | |
| Present-day | 244 | 205 | -39 | -16% | 205 | -39 | -16% | 208 | -36 | -15% | 206 | -38 | -16% |
| RCP2.6 2030 | 246 | 213 | -33 | -13% | 208 | -38 | -15% | 216 | -30 | -12% | 215 | -31 | -13% |
| RCP2.6 2040 | 255 | 218 | -37 | -15% | 212 | -43 | -17% | 218 | -37 | -15% | 220 | -35 | -14% |
| RCP2.6 2050 | 253 | 220 | -33 | -13% | 215 | -38 | -15% | 223 | -30 | -12% | 221 | -32 | -13% |
| **Days *T*_db_max_ ≥ 35 °C** | | | | | | | | | | | | | |
| Present-day | 51 | 22 | -29 | -57% | 23 | -28 | -55% | 29 | -22 | -43% | 23 | -28 | -55% |
| RCP2.6 2030 | 61 | 29 | -32 | -52% | 31 | -30 | -49% | 36 | -25 | -41% | 31 | -30 | -49% |
| RCP2.6 2040 | 69 | 34 | -35 | -51% | 41 | -28 | -41% | 46 | -23 | -33% | 38 | -31 | -45% |
| RCP2.6 2050 | 76 | 41 | -35 | -46% | 44 | -32 | -42% | 47 | -29 | -38% | 41 | -35 | -46% |
| **Days *WBGT*_max_ ≥28 °C** | | | | | | | | | | | | | |
| Present-day | 176 | 119 | -57 | -32% | 119 | -57 | -32% | 130 | -46 | -26% | 119 | -57 | -32% |
| RCP2.6 2030 | 174 | 133 | -41 | -24% | 133 | -41 | -24% | 143 | -31 | -18% | 136 | -38 | -22% |
| RCP2.6 2040 | 183 | 142 | -41 | -22% | 139 | -44 | -24% | 145 | -38 | -21% | 143 | -40 | -22% |
| RCP2.6 2050 | 187 | 144 | -43 | -23% | 145 | -42 | -22% | 150 | -37 | -20% | 145 | -42 | -22% |
| **Days *WBGT*_max_ ≥30 °C** | | | | | | | | | | | | | |
| Present-day | 99 | 44 | -55 | -56% | 38 | -61 | -62% | 44 | -55 | -56% | 44 | -55 | -56% |
| RCP2.6 2030 | 108 | 48 | -60 | -56% | 50 | -58 | -54% | 58 | -50 | -46% | 49 | -59 | -55% |
| RCP2.6 2040 | 123 | 57 | -66 | -54% | 58 | -65 | -53% | 62 | -61 | -50% | 58 | -65 | -53% |
| RCP2.6 2050 | 127 | 58 | -69 | -54% | 58 | -69 | -54% | 69 | -58 | -46% | 59 | -68 | -54% |

**Table 5.** Effect of passive cooling strategies relative to no intervention (Existing) on maximum indoor dry-bulb (*T*_db_max_), and wet-bulb globe temperatures (*WBGT*_max_) for annual working days (*n* = 293) for present-day and future decades under RCP4.5.

|  | **Existing** | **Green Roof** | | | **Shaded Roof** | | | **White Roof** | | | **Insulated White Roof** | | |
| --- | --- | --- | --- | --- | --- | --- | --- | --- | --- | --- | --- | --- | --- |
|  | # days | # days | Δ days | Δ % | # days | Δ days | Δ % | # days | Δ days | Δ % | # days | Δ days | Δ % |
| **Days *T*_db_max_ ≥ 30 °C** | | | | | | | | | | | | | |
| Present-day | 244 | 205 | -39 | -16% | 205 | -39 | -16% | 208 | -36 | -15% | 206 | -38 | -16% |
| RCP4.5 2030 | 249 | 220 | -29 | -12% | 215 | -34 | -14% | 223 | -26 | -10% | 221 | -28 | -11% |
| RCP4.5 2040 | 262 | 223 | -39 | -15% | 221 | -41 | -16% | 230 | -32 | -12% | 225 | -37 | -14% |
| RCP4.5 2050 | 261 | 234 | -27 | -10% | 231 | -30 | -11% | 231 | -30 | -11% | 235 | -26 | -10% |
| **Days *T*_db_max_ ≥ 35 °C** | | | | | | | | | | | | | |
| Present-day | 51 | 22 | -29 | -57% | 23 | -28 | -55% | 29 | -22 | -43% | 23 | -28 | -55% |
| RCP4.5 2030 | 70 | 31 | -39 | -56% | 32 | -38 | -54% | 39 | -31 | -44% | 31 | -39 | -56% |
| RCP4.5 2040 | 89 | 46 | -43 | -48% | 47 | -42 | -47% | 58 | -31 | -35% | 46 | -43 | -48% |
| RCP4.5 2050 | 95 | 48 | -47 | -49% | 50 | -45 | -47% | 65 | -30 | -32% | 48 | -47 | -49% |
| **Days *WBGT*_max_ ≥28 °C** | | | | | | | | | | | | | |
| Present-day | 176 | 119 | -57 | -32% | 119 | -57 | -32% | 130 | -46 | -26% | 119 | -57 | -32% |
| RCP4.5 2030 | 186 | 145 | -41 | -22% | 145 | -41 | -22% | 150 | -36 | -19% | 146 | -40 | -22% |
| RCP4.5 2040 | 191 | 150 | -41 | -21% | 146 | -45 | -24% | 153 | -38 | -20% | 152 | -39 | -20% |
| RCP4.5 2050 | 198 | 156 | -42 | -21% | 155 | -43 | -22% | 164 | -34 | -17% | 156 | -42 | -21% |
| **Days *WBGT*_max_ ≥30 °C** | | | | | | | | | | | | | |
| Present-day | 99 | 44 | -55 | -56% | 38 | -61 | -62% | 44 | -55 | -56% | 44 | -55 | -56% |
| RCP4.5 2030 | 117 | 60 | -57 | -49% | 60 | -57 | -49% | 65 | -52 | -44% | 61 | -56 | -48% |
| RCP4.5 2040 | 134 | 66 | -68 | -51% | 65 | -69 | -51% | 70 | -64 | -48% | 66 | -68 | -51% |
| RCP4.5 2050 | 142 | 80 | -62 | -44% | 80 | -62 | -44% | 83 | -59 | -42% | 82 | -60 | -42% |

**Table 6.** Effect of passive cooling strategies relative to no intervention (Existing) on maximum indoor dry-bulb (*T*_db_max_), and wet-bulb globe temperatures (*WBGT*_max_) for annual working days (*n* = 293) for present-day and future decades under RCP8.5.

|  | **Existing** | **Green Roof** | | | **Shaded Roof** | | | **White Roof** | | | **Insulated White Roof** | | |
| --- | --- | --- | --- | --- | --- | --- | --- | --- | --- | --- | --- | --- | --- |
|  | # days | # days | Δ days | Δ % | # days | Δ days | Δ % | # days | Δ days | Δ % | # days | Δ days | Δ % |
| **Days *T*_db_max_ ≥ 30 °C** | | | | | | | | | | | | | |
| Present-day | 244 | 205 | -39 | -16% | 205 | -39 | -16% | 208 | -36 | -15% | 206 | -38 | -16% |
| RCP8.5 2030 | 248 | 208 | -40 | -16% | 206 | -42 | -17% | 211 | -37 | -15% | 209 | -39 | -16% |
| RCP8.5 2040 | 257 | 224 | -33 | -13% | 221 | -36 | -14% | 231 | -26 | -10% | 227 | -30 | -12% |
| RCP8.5 2050 | 268 | 233 | -35 | -13% | 232 | -36 | -13% | 239 | -29 | -11% | 236 | -32 | -12% |
| **Days *T*_db_max_ ≥ 35 °C** | | | | | | | | | | | | | |
| Present-day | 51 | 22 | -29 | -57% | 23 | -28 | -55% | 29 | -22 | -43% | 23 | -28 | -55% |
| RCP8.5 2030 | 72 | 36 | -36 | -50% | 39 | -33 | -46% | 43 | -29 | -40% | 36 | -36 | -50% |
| RCP8.5 2040 | 99 | 52 | -47 | -47% | 57 | -42 | -42% | 65 | -34 | -34% | 52 | -47 | -47% |
| RCP8.5 2050 | 108 | 55 | -53 | -49% | 61 | -47 | -44% | 70 | -38 | -35% | 56 | -52 | -48% |
| **Days *WBGT*_max_ ≥28 °C** | | | | | | | | | | | | | |
| Present-day | 176 | 119 | -57 | -32% | 119 | -57 | -32% | 130 | -46 | -26% | 119 | -57 | -32% |
| RCP8.5 2030 | 188 | 138 | -50 | -27% | 137 | -51 | -27% | 143 | -45 | -24% | 140 | -48 | -26% |
| RCP8.5 2040 | 188 | 156 | -32 | -17% | 157 | -31 | -16% | 164 | -24 | -13% | 158 | -30 | -16% |
| RCP8.5 2050 | 199 | 168 | -31 | -16% | 166 | -33 | -17% | 171 | -28 | -14% | 170 | -29 | -15% |
| **Days *WBGT*_max_ ≥30 °C** | | | | | | | | | | | | | |
| Present-day | 99 | 44 | -55 | -56% | 38 | -61 | -62% | 44 | -55 | -56% | 44 | -55 | -56% |
| RCP8.5 2030 | 119 | 55 | -64 | -54% | 54 | -65 | -55% | 65 | -54 | -45% | 56 | -63 | -53% |
| RCP8.5 2040 | 136 | 68 | -68 | -50% | 70 | -66 | -49% | 83 | -53 | -39% | 68 | -68 | -50% |
| RCP8.5 2050 | 154 | 92 | -62 | -40% | 92 | -62 | -40% | 105 | -49 | -32% | 92 | -62 | -40% |

**Table 7.** Effect of passive cooling strategies relative to no intervention (Existing) on wet-bulb globe temperatures (*WBGT*) work rate categories for annual working hours (*n* = 3,708) for present-day and future decades under RCP2.6.

|  |  | **Existing** | **Green Roof** | | | **Shaded Roof** | | | **White Roof** | | | **Insulated White Roof** | | |
| --- | --- | --- | --- | --- | --- | --- | --- | --- | --- | --- | --- | --- | --- | --- |
| **WBGT** | **Decade** | # hours | # hours | Δ hours | Δ % | # hours | Δ hours | Δ % | # hours | Δ hours | Δ % | # hours | Δ hours | Δ % |
| **<23 °C** | Present-day | 745 | 988 | 243 | 33% | 1077 | 332 | 45% | 1037 | 292 | 39% | 972 | 227 | 30% |
|  | RCP2.6 2030 | 722 | 904 | 182 | 25% | 1023 | 301 | 42% | 980 | 258 | 36% | 894 | 172 | 24% |
|  | RCP2.6 2040 | 649 | 907 | 258 | 40% | 1007 | 358 | 55% | 959 | 310 | 48% | 882 | 233 | 36% |
|  | RCP2.6 2050 | 620 | 829 | 209 | 34% | 933 | 313 | 50% | 891 | 271 | 44% | 807 | 187 | 30% |
| **≥23-25 °C** | Present-day | 458 | 469 | 11 | 2% | 463 | 5 | 1% | 460 | 2 | 0% | 479 | 21 | 5% |
|  | RCP2.6 2030 | 456 | 549 | 93 | 20% | 497 | 41 | 9% | 499 | 43 | 9% | 550 | 94 | 21% |
|  | RCP2.6 2040 | 477 | 490 | 13 | 3% | 440 | -37 | -8% | 446 | -31 | -6% | 496 | 19 | 4% |
|  | RCP2.6 2050 | 455 | 484 | 29 | 6% | 448 | -7 | -2% | 447 | -8 | -2% | 491 | 36 | 8% |
| **≥25-26 °C** | Present-day | 252 | 324 | 72 | 29% | 302 | 50 | 20% | 279 | 27 | 11% | 312 | 60 | 24% |
|  | RCP2.6 2030 | 258 | 258 | 0 | 0% | 251 | -7 | -3% | 250 | -8 | -3% | 258 | 0 | 0% |
|  | RCP2.6 2040 | 243 | 222 | -21 | -9% | 232 | -11 | -5% | 234 | -9 | -4% | 227 | -16 | -7% |
|  | RCP2.6 2050 | 232 | 231 | -1 | 0% | 233 | 1 | 0% | 230 | -2 | -1% | 236 | 4 | 2% |
| **≥26-28 °C** | Present-day | 651 | 896 | 245 | 38% | 867 | 216 | 33% | 844 | 193 | 30% | 907 | 256 | 39% |
|  | RCP2.6 2030 | 596 | 791 | 195 | 33% | 798 | 202 | 34% | 750 | 154 | 26% | 787 | 191 | 32% |
|  | RCP2.6 2040 | 595 | 774 | 179 | 30% | 758 | 163 | 27% | 720 | 125 | 21% | 781 | 186 | 31% |
|  | RCP2.6 2050 | 588 | 807 | 219 | 37% | 802 | 214 | 36% | 766 | 178 | 30% | 799 | 211 | 36% |
| **≥28-30 °C** | Present-day | 981 | 757 | -224 | -23% | 764 | -217 | -22% | 814 | -167 | -17% | 761 | -220 | -22% |
|  | RCP2.6 2030 | 909 | 921 | 12 | 1% | 855 | -54 | -6% | 893 | -16 | -2% | 927 | 18 | 2% |
|  | RCP2.6 2040 | 879 | 933 | 54 | 6% | 917 | 38 | 4% | 933 | 54 | 6% | 931 | 52 | 6% |
|  | RCP2.6 2050 | 892 | 929 | 37 | 4% | 885 | -7 | -1% | 909 | 17 | 2% | 939 | 47 | 5% |
| **≥30-33 °C** | Present-day | 601 | 274 | -327 | -54% | 235 | -366 | -61% | 274 | -327 | -54% | 277 | -324 | -54% |
|  | RCP2.6 2030 | 733 | 285 | -448 | -61% | 284 | -449 | -61% | 336 | -397 | -54% | 292 | -441 | -60% |
|  | RCP2.6 2040 | 816 | 381 | -435 | -53% | 353 | -463 | -57% | 412 | -404 | -50% | 390 | -426 | -52% |
|  | RCP2.6 2050 | 836 | 428 | -408 | -49% | 406 | -430 | -51% | 454 | -382 | -46% | 435 | -401 | -48% |
| **>33 °C** | Present-day | 20 | 0 | -20 | -100% | 0 | -20 | -100% | 0 | -20 | -100% | 0 | -20 | -100% |
|  | RCP2.6 2030 | 34 | 0 | -34 | -100% | 0 | -34 | -100% | 0 | -34 | -100% | 0 | -34 | -100% |
|  | RCP2.6 2040 | 49 | 1 | -48 | -98% | 1 | -48 | -98% | 4 | -45 | -92% | 1 | -48 | -98% |
|  | RCP2.6 2050 | 85 | 0 | -85 | -100% | 1 | -84 | -99% | 11 | -74 | -87% | 1 | -84 | -99% |

*Note:* Discreate categories based around work limits for acclimatised workers as per ISO [1], ACHIH [2], and NIOSH [3]. Categories align approximately with the following work rates: WBGT 23-25 °C = very heavy work (407-580 W), 25-26 °C = heavy work (350-468 W), 26-28 °C = moderate work (234-407 W), 28-30 °C = light work (233-360 W), 30-33 °C = resting (117-233 W).

**Table 8.** Effect of passive cooling strategies relative to no intervention (Existing) on wet-bulb globe temperatures (*WBGT*) work rate categories for annual working hours (*n* = 3,708) for present-day and future decades under RCP4.5.

|  |  | **Existing** | **Green Roof** | | | **Shaded Roof** | | | **White Roof** | | | **Insulated White Roof** | | |
| --- | --- | --- | --- | --- | --- | --- | --- | --- | --- | --- | --- | --- | --- | --- |
| **WBGT** | **Decade** | # hours | # hours | Δ hours | Δ % | # hours | Δ hours | Δ % | # hours | Δ hours | Δ % | # hours | Δ hours | Δ % |
| **<23 °C** | Present-day | 745 | 988 | 243 | 33% | 1077 | 332 | 45% | 1037 | 292 | 39% | 972 | 227 | 30% |
|  | RCP4.5 2030 | 664 | 847 | 183 | 28% | 966 | 302 | 45% | 920 | 256 | 39% | 825 | 161 | 24% |
|  | RCP4.5 2040 | 597 | 791 | 194 | 32% | 883 | 286 | 48% | 833 | 236 | 40% | 759 | 162 | 27% |
|  | RCP4.5 2050 | 514 | 695 | 181 | 35% | 812 | 298 | 58% | 818 | 304 | 59% | 665 | 151 | 29% |
| **≥23-25 °C** | Present-day | 458 | 469 | 11 | 2% | 463 | 5 | 1% | 460 | 2 | 0% | 479 | 21 | 5% |
|  | RCP4.5 2030 | 416 | 474 | 58 | 14% | 426 | 10 | 2% | 418 | 2 | 0% | 479 | 63 | 15% |
|  | RCP4.5 2040 | 406 | 487 | 81 | 20% | 463 | 57 | 14% | 472 | 66 | 16% | 501 | 95 | 23% |
|  | RCP4.5 2050 | 423 | 468 | 45 | 11% | 432 | 9 | 2% | 414 | -9 | -2% | 474 | 51 | 12% |
| **≥25-26 °C** | Present-day | 252 | 324 | 72 | 29% | 302 | 50 | 20% | 279 | 27 | 11% | 312 | 60 | 24% |
|  | RCP4.5 2030 | 241 | 259 | 18 | 7% | 262 | 21 | 9% | 266 | 25 | 10% | 268 | 27 | 11% |
|  | RCP4.5 2040 | 262 | 237 | -25 | -10% | 251 | -11 | -4% | 239 | -23 | -9% | 240 | -22 | -8% |
|  | RCP4.5 2050 | 219 | 291 | 72 | 33% | 285 | 66 | 30% | 289 | 70 | 32% | 297 | 78 | 36% |
| **≥26-28 °C** | Present-day | 651 | 896 | 245 | 38% | 867 | 216 | 33% | 844 | 193 | 30% | 907 | 256 | 39% |
|  | RCP4.5 2030 | 641 | 814 | 173 | 27% | 792 | 151 | 24% | 764 | 123 | 19% | 810 | 169 | 26% |
|  | RCP4.5 2040 | 593 | 757 | 164 | 28% | 740 | 147 | 25% | 713 | 120 | 20% | 763 | 170 | 29% |
|  | RCP4.5 2050 | 567 | 627 | 60 | 11% | 627 | 60 | 11% | 630 | 63 | 11% | 636 | 69 | 12% |
| **≥28-30 °C** | Present-day | 981 | 757 | -224 | -23% | 764 | -217 | -22% | 814 | -167 | -17% | 761 | -220 | -22% |
|  | RCP4.5 2030 | 930 | 969 | 39 | 4% | 938 | 8 | 1% | 958 | 28 | 3% | 973 | 43 | 5% |
|  | RCP4.5 2040 | 864 | 987 | 123 | 14% | 950 | 86 | 10% | 984 | 120 | 14% | 996 | 132 | 15% |
|  | RCP4.5 2050 | 884 | 1074 | 190 | 21% | 1026 | 142 | 16% | 1001 | 117 | 13% | 1074 | 190 | 21% |
| **≥30-33 °C** | Present-day | 601 | 274 | -327 | -54% | 235 | -366 | -61% | 274 | -327 | -54% | 277 | -324 | -54% |
|  | RCP4.5 2030 | 772 | 345 | -427 | -55% | 324 | -448 | -58% | 381 | -391 | -51% | 353 | -419 | -54% |
|  | RCP4.5 2040 | 916 | 446 | -470 | -51% | 418 | -498 | -54% | 462 | -454 | -50% | 446 | -470 | -51% |
|  | RCP4.5 2050 | 1012 | 551 | -461 | -46% | 520 | -492 | -49% | 552 | -460 | -45% | 560 | -452 | -45% |
| **>33 °C** | Present-day | 20 | 0 | -20 | -100% | 0 | -20 | -100% | 0 | -20 | -100% | 0 | -20 | -100% |
|  | RCP4.5 2030 | 44 | 0 | -44 | -100% | 0 | -44 | -100% | 1 | -43 | -98% | 0 | -44 | -100% |
|  | RCP4.5 2040 | 70 | 3 | -67 | -96% | 3 | -67 | -96% | 5 | -65 | -93% | 3 | -67 | -96% |
|  | RCP4.5 2050 | 89 | 2 | -87 | -98% | 6 | -83 | -93% | 4 | -85 | -96% | 2 | -87 | -98% |

*Note:* Discreate categories based around work limits for acclimatised workers as per ISO [1], ACHIH [2], and NIOSH [3]. Categories align approximately with the following work rates: WBGT 23-25 °C = very heavy work (407-580 W), 25-26 °C = heavy work (350-468 W), 26-28 °C = moderate work (234-407 W), 28-30 °C = light work (233-360 W), 30-33 °C = resting (117-233 W).

**Table 9.** Effect of passive cooling strategies relative to no intervention (Existing) on wet-bulb globe temperatures (*WBGT*) work rate categories for annual working hours (*n* = 3,708) for present-day and future decades under RCP8.5.

|  |  | **Existing** | **Green Roof** | | | **Shaded Roof** | | | **White Roof** | | | **Insulated White Roof** | | |
| --- | --- | --- | --- | --- | --- | --- | --- | --- | --- | --- | --- | --- | --- | --- |
| **WBGT** | **Decade** | # hours | # hours | Δ hours | Δ % | # hours | Δ hours | Δ % | # hours | Δ hours | Δ % | # hours | Δ hours | Δ % |
| **<23 °C** | Present-day | 745 | 988 | 243 | 33% | 1077 | 332 | 45% | 1037 | 292 | 39% | 972 | 227 | 30% |
|  | RCP8.5 2030 | 636 | 875 | 239 | 38% | 959 | 323 | 51% | 919 | 283 | 44% | 845 | 209 | 33% |
|  | RCP8.5 2040 | 564 | 751 | 187 | 33% | 858 | 294 | 52% | 818 | 254 | 45% | 720 | 156 | 28% |
|  | RCP8.5 2050 | 453 | 634 | 181 | 40% | 750 | 297 | 66% | 700 | 247 | 55% | 607 | 154 | 34% |
| **≥23-25 °C** | Present-day | 458 | 469 | 11 | 2% | 463 | 5 | 1% | 460 | 2 | 0% | 479 | 21 | 5% |
|  | RCP8.5 2030 | 482 | 456 | -26 | -5% | 447 | -35 | -7% | 447 | -35 | -7% | 470 | -12 | -2% |
|  | RCP8.5 2040 | 418 | 457 | 39 | 9% | 415 | -3 | -1% | 414 | -4 | -1% | 478 | 60 | 14% |
|  | RCP8.5 2050 | 412 | 447 | 35 | 8% | 436 | 24 | 6% | 429 | 17 | 4% | 459 | 47 | 11% |
| **≥25-26 °C** | Present-day | 252 | 324 | 72 | 29% | 302 | 50 | 20% | 279 | 27 | 11% | 312 | 60 | 24% |
|  | RCP8.5 2030 | 240 | 251 | 11 | 5% | 245 | 5 | 2% | 243 | 3 | 1% | 251 | 11 | 5% |
|  | RCP8.5 2040 | 236 | 275 | 39 | 17% | 283 | 47 | 20% | 289 | 53 | 22% | 267 | 31 | 13% |
|  | RCP8.5 2050 | 233 | 307 | 74 | 32% | 272 | 39 | 17% | 272 | 39 | 17% | 298 | 65 | 28% |
| **≥26-28 °C** | Present-day | 651 | 896 | 245 | 38% | 867 | 216 | 33% | 844 | 193 | 30% | 907 | 256 | 39% |
|  | RCP8.5 2030 | 603 | 822 | 219 | 36% | 825 | 222 | 37% | 779 | 176 | 29% | 825 | 222 | 37% |
|  | RCP8.5 2040 | 587 | 704 | 117 | 20% | 686 | 99 | 17% | 630 | 43 | 7% | 698 | 111 | 19% |
|  | RCP8.5 2050 | 540 | 572 | 32 | 6% | 575 | 35 | 6% | 572 | 32 | 6% | 584 | 44 | 8% |
| **≥28-30 °C** | Present-day | 981 | 757 | -224 | -23% | 764 | -217 | -22% | 814 | -167 | -17% | 761 | -220 | -22% |
|  | RCP8.5 2030 | 909 | 984 | 75 | 8% | 931 | 22 | 2% | 952 | 43 | 5% | 991 | 82 | 9% |
|  | RCP8.5 2040 | 855 | 1027 | 172 | 20% | 992 | 137 | 16% | 1001 | 146 | 17% | 1045 | 190 | 22% |
|  | RCP8.5 2050 | 812 | 1028 | 216 | 27% | 1000 | 188 | 23% | 973 | 161 | 20% | 1030 | 218 | 27% |
| **≥30-33 °C** | Present-day | 601 | 274 | -327 | -54% | 235 | -366 | -61% | 274 | -327 | -54% | 277 | -324 | -54% |
|  | RCP8.5 2030 | 795 | 320 | -475 | -60% | 301 | -494 | -62% | 367 | -428 | -54% | 326 | -469 | -59% |
|  | RCP8.5 2040 | 946 | 491 | -455 | -48% | 474 | -472 | -50% | 552 | -394 | -42% | 497 | -449 | -47% |
|  | RCP8.5 2050 | 1109 | 699 | -410 | -37% | 658 | -451 | -41% | 737 | -372 | -34% | 708 | -401 | -36% |
| **>33 °C** | Present-day | 20 | 0 | -20 | -100% | 0 | -20 | -100% | 0 | -20 | -100% | 0 | -20 | -100% |
|  | RCP8.5 2030 | 43 | 0 | -43 | -100% | 0 | -43 | -100% | 1 | -42 | -98% | 0 | -43 | -100% |
|  | RCP8.5 2040 | 102 | 3 | -99 | -97% | 0 | -102 | -100% | 4 | -98 | -96% | 3 | -99 | -97% |
|  | RCP8.5 2050 | 149 | 21 | -128 | -86% | 17 | -132 | -89% | 25 | -124 | -83% | 22 | -127 | -85% |

*Note:* Discreate categories based around work limits for acclimatised workers as per ISO [1], ACHIH [2], and NIOSH [3]. Categories align approximately with the following work rates: WBGT 23-25 °C = very heavy work (407-580 W), 25-26 °C = heavy work (350-468 W), 26-28 °C = moderate work (234-407 W), 28-30 °C = light work (233-360 W), 30-33 °C = resting (117-233 W).


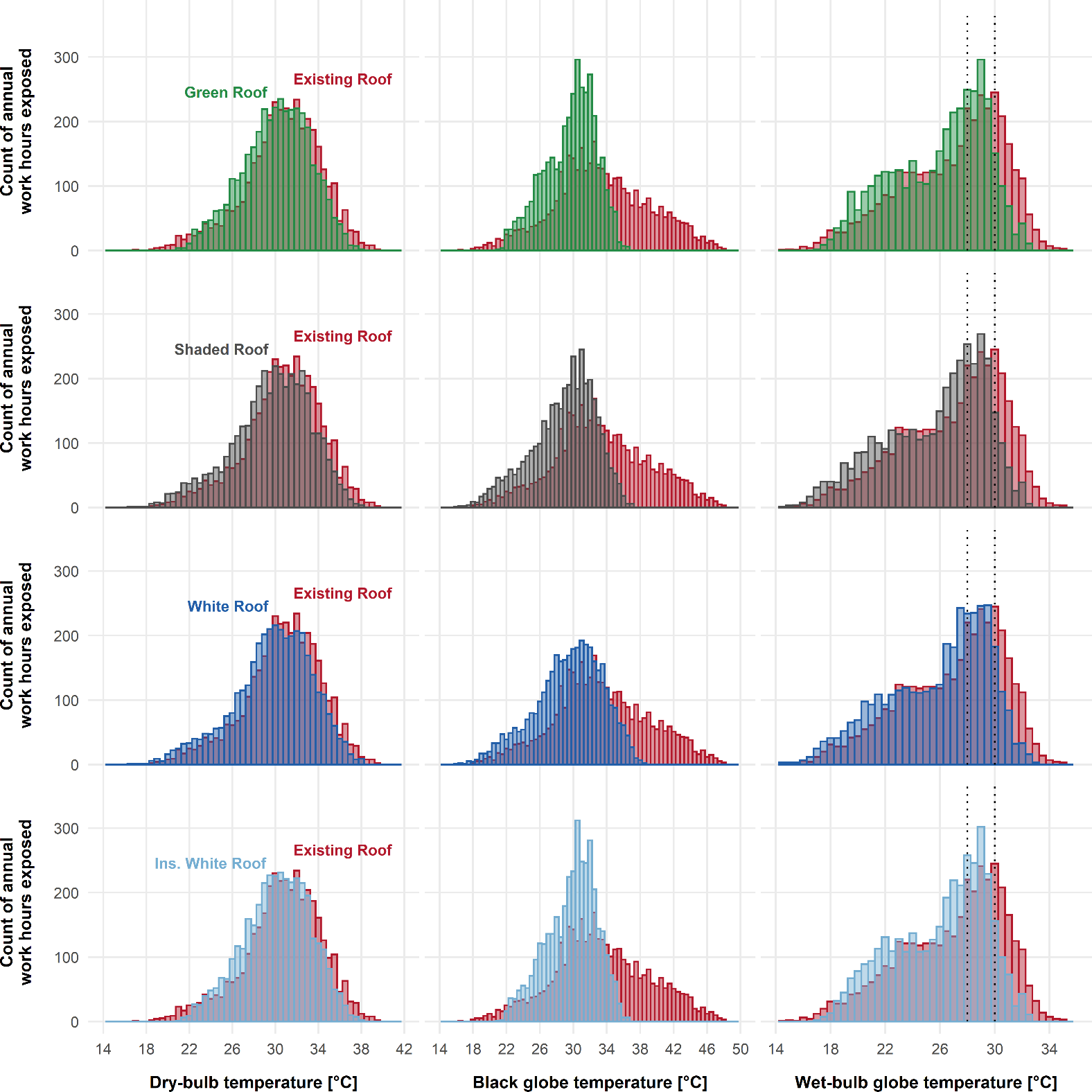


**Figure 2.** 2030 (RCP8.5) annual work hour distributions of indoor dry-bulb, black globe, and wet-bulb globe temperatures for different passive cooling strategies. Wet-bulb globe temperature thresholds of 28 °C and 30 °C (black dotted lines).
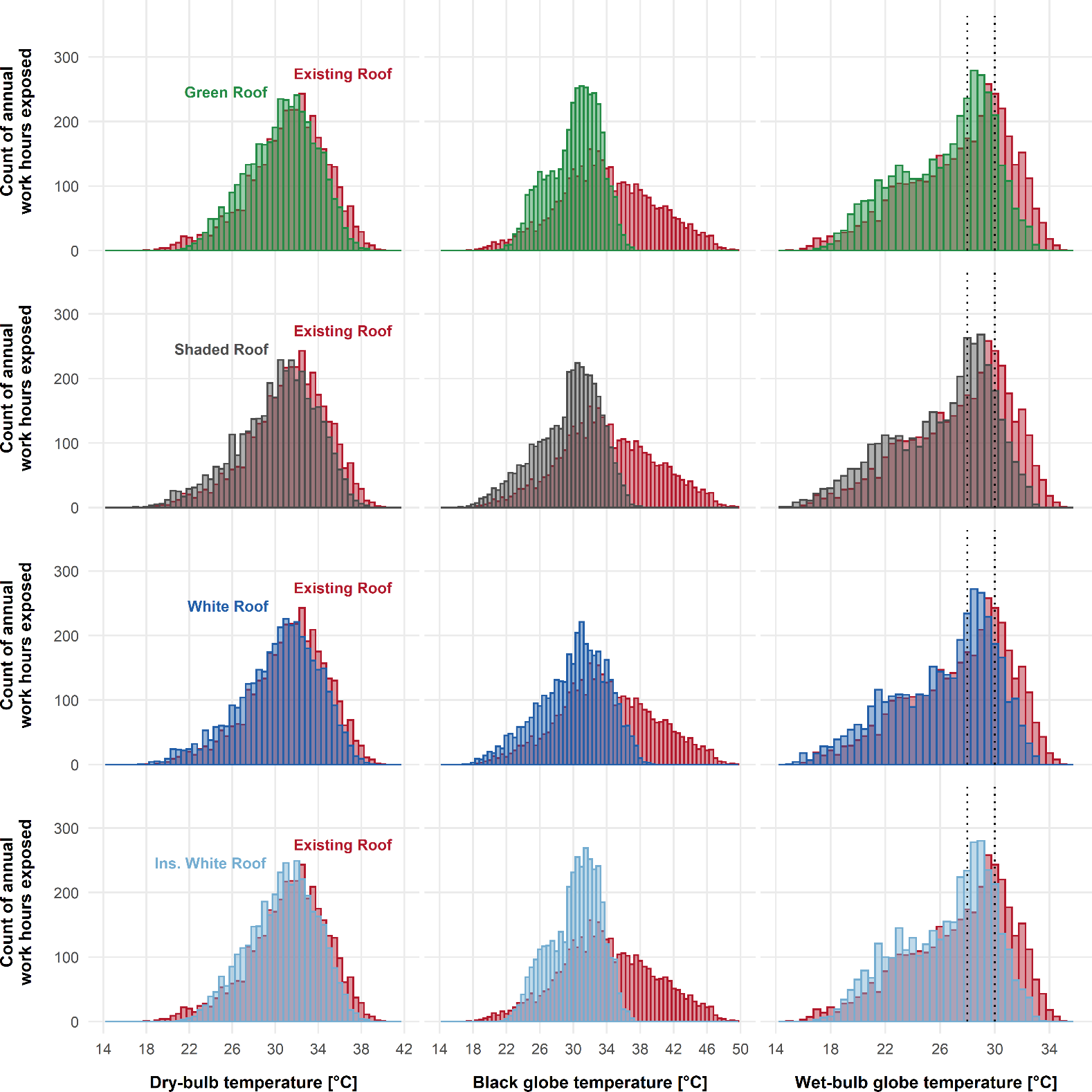


**Figure 3.** 2040 (RCP8.5) annual work hour distributions of indoor dry-bulb, black globe, and wet-bulb globe temperatures for different passive cooling strategies. Wet-bulb globe temperature thresholds of 28 °C and 30 °C (black dotted lines).


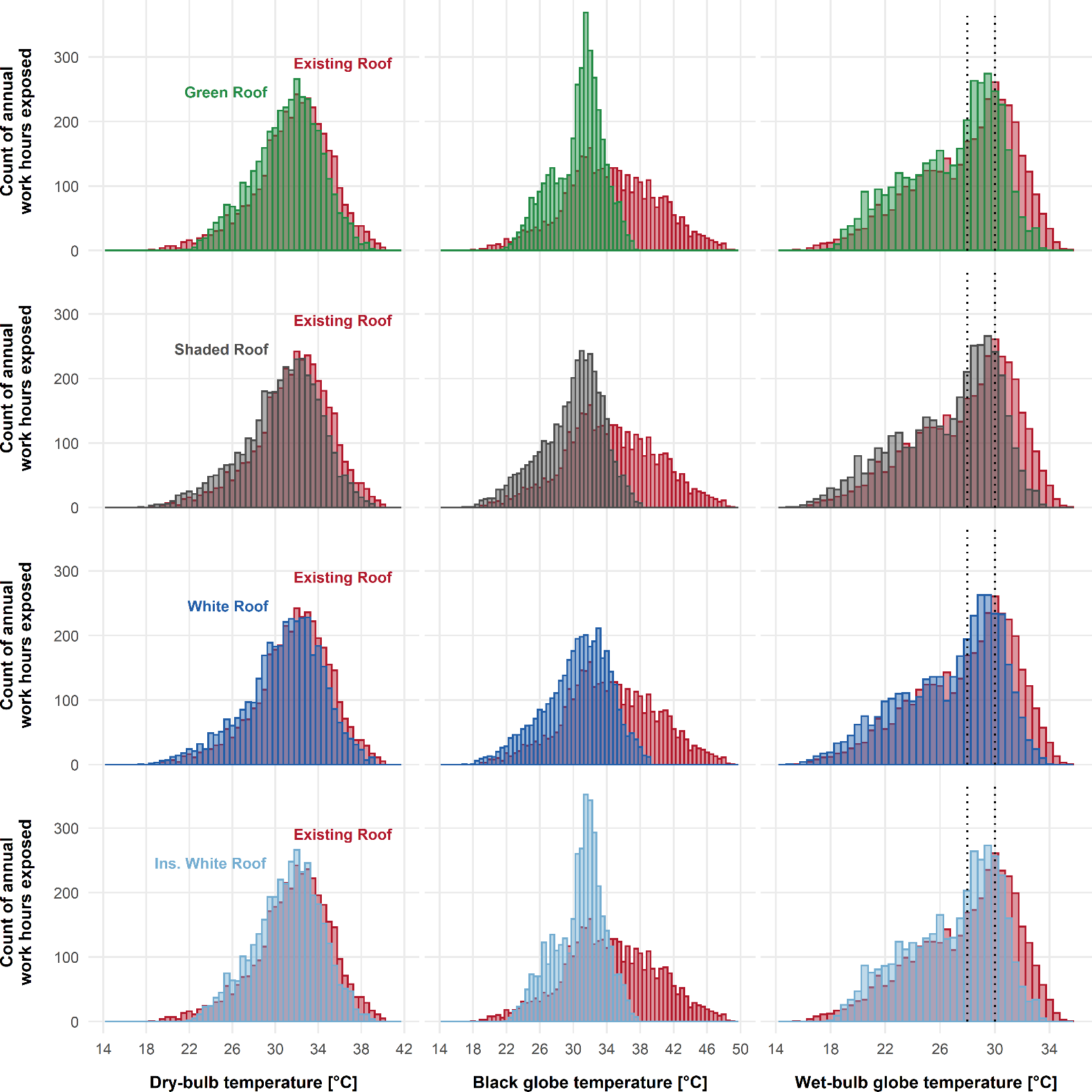


**Figure 4.** 2050 (RCP8.5) annual work hour distributions of indoor dry-bulb, black globe, and wet-bulb globe temperatures for different passive cooling strategies. Wet-bulb globe temperature thresholds of 28 °C and 30 °C (black dotted lines).

*4. References*

[1] ISO (2017). Ergonomics of the thermal environment - Assessment of heat stress using the WBGT (wet bulb globe temperature) index - ISO 7243:2017. Geneva, Switzerland: ISO.

[2] ACGIH (2018). 2019 threshold limit values (TLVs) and biological exposure indices (BEIs) Cincinnati, OH: ACGIH.

[3] NIOSH (2016). NIOSH criteria for a recommended standard: occupational exposure to heat and hot environments. Cincinnati, OH: U.S. Department of Health and Human Services, Centers for Disease Control and Prevention (CDC), National Institute for Occupational Safety and Health, DHHS (NIOSH).
